# CircUSP1 as a novel marker promotes gastric cancer progression via stabilizing HuR to upregulate USP1 and Vimentin

**DOI:** 10.1038/s41388-024-02968-8

**Published:** 2024-02-16

**Authors:** Rong Li, Junyi Wang, Zhenfan Xie, Xinyu Tian, Jie Hou, Dongli Wang, Hui Qian, Han Shen, Wenrong Xu

**Affiliations:** 1 Department of Laboratory Medicine, Nanjing Drum Tower Hospital, the Affiliated Hospital of Nanjing University Medical School, Nanjing University, 321 Zhongshan Road, Nanjing, Jiangsu 210008 China; 2 Jiangsu Key Laboratory of Medical Science and Laboratory Medicine, School of Medicine, Jiangsu University, 301 Xuefu Road, Zhenjiang, Jiangsu 212013 China; 3 Centre of Clinical Laboratory, the First Affiliated Hospital of Soochow University, 899 Pinghai Road, Suzhou, Jiangsu 215006 China; 4 Department of Clinical Laboratory, People’s Hospital of Yangzhong City, 235 Yangzi Middle Road, Zhenjiang, Jiangsu 212200 China

**Keywords:** Gastric cancer, Diagnostic markers, Oncogenes

## Abstract

Circular RNAs (circRNAs) play a crucial role in regulating various tumors. However, their biological functions and mechanisms in gastric cancer (GC) have not been well understood. Here, we discovered a stable cytoplasmic circRNA named circUSP1 (hsa_circ_000613) in GC. CircUSP1 upregulation in GC tissues was correlated with tumor size and differentiation. We observed that circUSP1 promoted GC growth and metastasis. Mechanistically, circUSP1 mainly interacted with the RRM1 domain of an RNA-binding protein (RBP) called HuR, stabilizing its protein level by inhibiting β-TrCP-mediated ubiquitination degradation. The oncogenic properties of HuR mediated promotive effects of circUSP1 in GC progression. Moreover, we identified USP1 and Vimentin as downstream targets of HuR in post-transcriptional regulation, mediating the effects of circUSP1. The parent gene USP1 also enhanced the viability and mobility of GC cells. Additionally, tissue-derived circUSP1 could serve as an independent prognostic factor for GC, while plasma-derived circUSP1 showed promise as a diagnostic biomarker, outperforming conventional markers including serum alpha-fetoprotein (AFP), carcinoembryonic antigen (CEA) and carbohydrate antigen 199 (CA19-9). Our study highlights that circUSP1 promotes GC progression by binding to and stabilizing oncogenic HuR, thereby facilitating the upregulation of USP1 and Vimentin at the post-transcriptional level. These findings suggest that circUSP1 could be a potential therapeutic target and a diagnostic and prognostic biomarker for GC.

## Introduction

Gastric cancer (GC) is the fifth most common cancer and the fourth leading cause of cancer-related deaths worldwide [1]. *Helicobacter pylori* infection, diet, exogenous chemicals, intragastric synthesis of carcinogens and genetic factors are known risk factors for gastric tumorigenesis but the exact mechanisms are not yet fully understood [2]. Since early-stage GC often presents without noticeable symptoms and lacks reliable biomarkers, most patients are diagnosed at the advanced stage with a five-year survival rate lower than 30% [3]. Additionally, current treatments such as radiotherapy and chemotherapy have limited effectiveness due to systemic cytotoxicity and drug resistance [4]. Therefore, there is an urgent need to gain a deeper understanding of the molecular mechanisms involved in GC progression and to discover new biomarkers and therapeutic targets to improve the prognosis of GC patients.

Circular RNAs (circRNAs) are a new type of endogenous RNAs characterized by a unique circular structure. They are generated by the back-splicing process of precursor mRNA (pre-mRNA), in which the 3’ splice acceptor site is joined to the 5’ donor site to form a covalently closed loop [5]. Recently, circRNAs have been discovered in various human tissues and body liquids, displaying remarkable stability, abundance [6], evolutionary conservation [7] and tissue/developmental-stage-specific expression pattern [8–10]. Moreover, numerous circRNAs are dysregulated in various tumors and participate in tumor proliferation, apoptosis, migration and invasion [11–13]. Cytoplasmic circRNAs primarily function as microRNA (miRNA) sponges [14], serve as templates for protein translation [15], or interact with RNA-binding proteins (RBPs) as sponges, decoys, scaffolds and recruiters [16], while nuclear circRNAs are involved in gene transcription regulation [17]. These properties confer circRNAs a great potential to be promising biomarkers and novel therapeutic targets for cancer. In GC, a growing number of circRNAs have been identified as crucial regulators in tumor progression [18]. For example, circURI1 suppresses GC metastasis by interacting with hnRNPM to modulate alternative splicing of migration-associated genes [19]. Oncogenic circOSBPL10 acts as a sponge for miR-136-5p to enhance the effects of WNT2 in GC growth and metastasis and serves as a new prognostic marker [20]. CircAXIN1 promotes GC progression by producing the AXIN1-295aa protein which activates the Wnt signaling pathway and could potentially be targeted for therapy [21]. Therefore, further investigation into circRNAs associated with GC would provide valuable insights into GC pathogenesis and identify new diagnostic and treatment strategies.

In this study, we have identified a new circRNA transcribed from the USP1 gene named circUSP1 (circBase ID: hsa_circ_000613) in GC and explored its biological roles, molecular mechanism and potential applications in clinical settings. Our findings reveal that circUSP1 plays a crucial role in promoting cancer by interacting with and stabilizing HuR protein, leading to an increase of USP1 and Vimentin at the post-transcriptional level. Increased circUSP1 in tumor tissues is associated with a poor prognosis of GC patients. Furthermore, we have found that circUSP1 can serve as a diagnostic biomarker for GC when detected in plasma samples. Our study provides new insight into the mechanisms involved in GC progression and identifies circUSP1 as a potential biomarker and therapeutic target for GC.

## Results

### Upregulation of circUSP1 in GC tissues and cells predicts poor prognosis

**Fig. 1:**
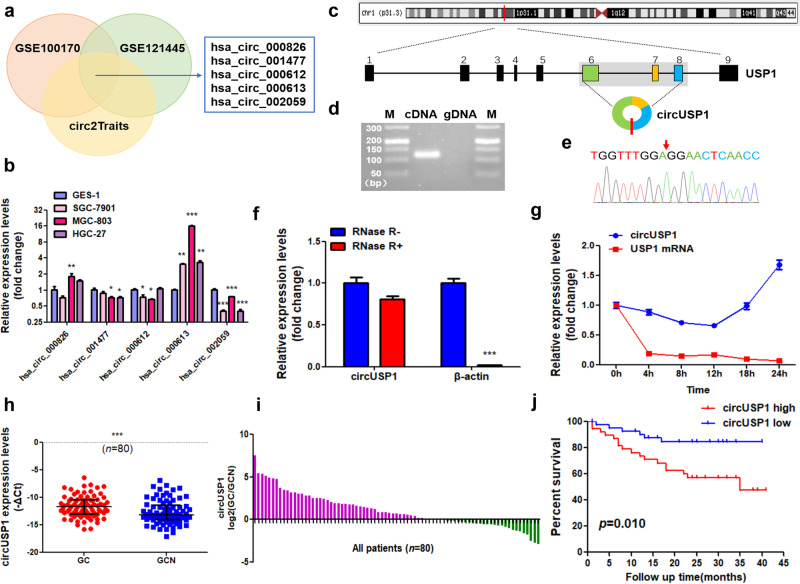
Upregulation of circUSP1 in GC tissues and cells predicts poor prognosis. **a** GEO datasets (GSE100170 and GSE121445) and circ2Traits were downloaded for integrated analysis of GC-related circRNAs. The common dysregulated circRNA are listed. **b** RT-qPCR analysis of five potential GC-related circRNA levels in GC and GES-1 cell lines. *p < 0.05, **p < 0.01, ***p < 0.001 versus GES-1 group, *n* = 3 [one-way ANOVA followed by Dunnett’s post-hoc test]. **c** The annotated region in USP1 gene for the formation of circUSP1 was shown according to UCSC Genome database (http://genome.ucsc.edu). **d** Agarose gel electrophoresis analysis of circUSP1 in cDNA and gDNA of MGC-803 cells. M represents DNA marker. **e** The head-to-tail splicing of circUSP1 was confirmed by Sanger sequencing. **f** RT-qPCR analysis of circUSP1 and β-actin mRNA levels in MGC-803 cells with or without RNase R treatment. ***p < 0.001 versus RNase R- group, *n* = 3 [Student’s t-test]. **g** RT-qPCR analysis of circUSP1 and USP1 mRNA levels in MGC-803 after actinomycin D treatment. **h** Comparison of circUSP1 level in paired GC tissues and adjacent normal tissues (GCN). ***p < 0.001 versus GC group, *n* = 80 [Wilcoxon signed-rank test]. **i** RT-qPCR analysis of circUSP1 level in GC and adjacent normal (GCN) tissues from 80 patients. Data are presented as log2 fold change and the red (green) bar represents upregulation (downregulation). **j** Kaplan-Meier analysis of correlation of circUSP1 level in GC tissues with overall survival of GC patients.

To discover new circRNAs involved in the development of GC, we conducted a comprehensive search of circRNA microarray data (GSE100170 and GSE121445) from NCBI Gene Expression Omnibus (GEO, https://www.ncbi.nlm.nih.gov/geo/) and GC related circRNAs in circ2Traits (http://gyanxet-bata.com/circdb/). After analyzing these data, several dysregulated circRNAs that were highly associated with GC progression were identified including hsa_circ_000826, hsa_circ_001477, hsa_circ_000612, hsa_circ_000613 and hsa_circ_002059 (Fig. 1a). To validate their expression levels in GC cells, we performed RT-qPCR using specific divergent primers (Supplementary Fig. S1a). Our results indicated that only hsa_circ_000613 was significantly upregulated in GC cells compared to normal GES-1 cells (Fig. 1b) and thus chosen for further study. Hsa_circ_000613 (chr1:62910408-62914337, hsa_circ_0000080) is generated from back-spliced exons 6, 7 and 8 of gene USP1 with a length of 1065 bp (Fig. 1c), which we termed as circUSP1. RT-qPCR results revealed that circUSP1 could be amplified from cDNA but not gDNA of GC cells (Fig. 1d). Meanwhile, melt curve analysis and Sanger sequencing verified the specificity and the back-spliced junction sequence of circUSP1 (Supplementary Fig. S1b, Fig. 1e). To assess its stability, we conducted RNase R assays and found that circUSP1 exhibited high resistance to exonuclease degradation, while linear mRNA was digested (Fig. 1f). Additionally, we treated GC cells with transcription inhibitor actinomycin D to monitor RNA degradation. The half-life of circUSP1 was over 12 h, whereas its counterpart mRNA USP1 had a shorter half-life of less than 4 hours (Fig. 1g), highlighting the high stability of circUSP1. These results indicate that circUSP1 is a newly discovered circular RNA in GC.

Additionally, we examined the expression level of circUSP1 in human GC tissues. It was markedly upregulated in GC tissues compared with adjacent normal tissues (Fig. 1h). About 65% (52/80) of GC patients exhibited higher levels of circUSP1 in GC tissues (Fig. 1i). CircUSP1 level was positively related to tumor size and negatively associated with tumor differentiation (Table 1). Furthermore, we divided GC patients into two groups (circUSP1 high and circUSP1 low) according to the average level of circUSP1. Patients with high circUSP1 levels exhibited shorter post-operative survival time (Fig. 1j). Cox regression analysis indicated that lymphatic metastasis, distal metastasis, TNM stage, tumor size and circUSP1 level were significantly associated with the overall survival (OS) of GC patients, among which tumor size and circUSP1 level were independent predictors (Table 2). The results above suggest that circUSP1 is highly expressed in GC tissues and could be used as an independent prognostic factor.

**Table 1:** Relationship of circUSP1 expression level (-△Ct) in tumor tissues with clinicopathological factors of GC patients.

Characteristics	No.of cases	circUSP1	
		Mean ± SD	*p* value
Age			
<60	20	−11.11 ± 2.01	0.179
≥60	60	−11.79 ± 1.93	
Gender			
Male	59	−11.74 ± 1.90	0.346
Female	21	−11.27 ± 2.11	
Tumor size			
<5 cm	38	−12.24 ± 1.60	0.006*
≥5 cm	42	−11.06 ± 2.09	
Differentiation			
Poor	44	−11.06 ± 2.00	0.010*
Moderate	34	−12.23 ± 1.67	
Well	2	−13.57 ± 2.00	
Lymphatic metastasis			
N0	22	−11.71 ± 1.51	0.980
N1	7	−11.44 ± 2.17	
N2	10	−11.76 ± 2.16	
N3	41	−11.57 ± 2.14	
Distal metastasis			
Absent	76	−11.66 ± 1.91	0.379
Present	4	−10.78 ± 2.90	
TNM stage			
I	6	−11.57 ± 1.21	0.839
II	16	−11.95 ± 1.65	
III	52	−11.58 ± 2.07	
IV	6	−11.11 ± 2.58	
Invasion depth			
T1	3	−11.12 ± 1.43	0.685
T2	5	−12.37 ± 1.21	
T3	7	−12.12 ± 1.09	
T4	65	−11.53 ± 2.09	

Bold values indicate significance at *p* < 0.05.

**Table 2:** Univariate and multivariate Cox regression analyses of potential prognostic variables in GC patients.

Variables	Subset	Hazard ratio for OS (95%CI)	*p* value
**Univariate analysis (*n*** = **80)**			
Age (years)	<60 vs ≥60	–	0.685
Gender	Male vs Female	–	0.932
Invasion depth	T1 + T2 vs T3 + T4	–	0.221
Tumor differentiation	Poor vs Moderate/Well	–	0.092
Lymphatic metastasis	N0 + N1 vs N2 + N3	1.512 (1.023–2.235)	0.038*
Distal metastasis	Absent vs Present	5.446 (1.589–18.666)	0.007*
TNM stage	I + II vs III + IV	2.825 (1.322–6.037)	0.007*
Tumor size (cm)	<5 vs ≥5	3.297 (1.295–8.395)	0.012*
circUSP1 level	Low vs High	3.213 (1.260–8.192)	0.015*
**Multivariate analysis (*n*** = **80)**			
Lymphatic metastasis	N0 + N1 vs N2 + N3	–	0.490
Distal metastasis	Absent vs present	–	0.113
TNM stage	I + II vs III + IV	–	0.501
Tumor size (cm)	< 5 vs ≥ 5	2.974 (1.130–7.826)	0.027*
circUSP1 level	Low vs High	2.777 (1.051–7.336)	0.039*

Bold values indicate significance at *p* < 0.05.

### CircUSP1 exerts oncogenic effects in GC

**Fig. 2:**
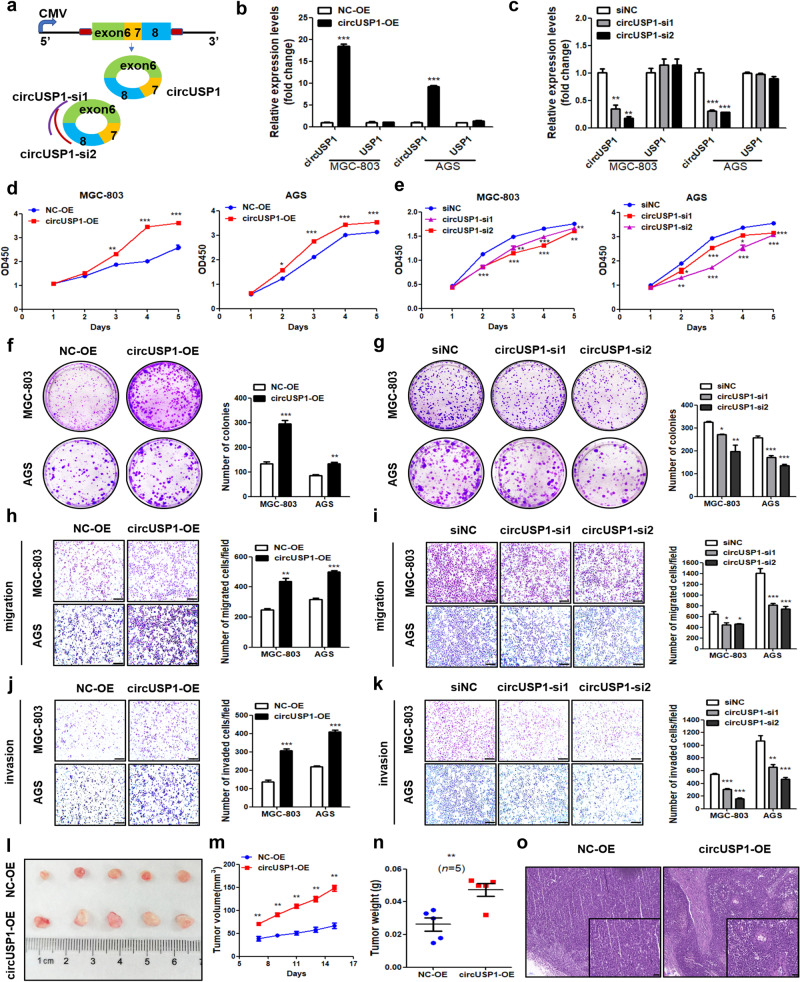
CircUSP1 exerts oncogenic effects in GC. **a** Schematic illustration of the circUSP1-overexpressing vector and two specific siRNAs. The flanking circularization elements are marked in red and siRNAs were designed to target the back-spliced junction of circUSP1. **b**, **c** RT-qPCR analyses of circUSP1 and its parent gene USP1 levels after circUSP1 overexpression and knockdown in MGC-803 and AGS cell lines. ***p < 0.001 versus NC-OE group, *n* = 3 [Student’s t-test]. **p < 0.01, ***p < 0.001 versus siNC group, *n* = 3 [one-way ANOVA followed by Dunnett’s post-hoc test]. **d**, **e** CCK8 assays of GC cells with circUSP1 overexpression and knockdown. *p < 0.05, **p < 0.01, ***p < 0.001 versus NC-OE group, *n* = 3 [Student’s t-test]. *p < 0.05, **p < 0.01, ***p < 0.001 versus siNC group, *n* = 3 [one-way ANOVA followed by Dunnett’s post-hoc test]. Colony formation assays (**f**, **g**), migration (**h**, **i**) and invasion (**j**, **k**) assays of GC cells with circUSP1 overexpression and knockdown. The scale bar indicates 200 μm. The right bar graphs show the quantitative comparison of colony numbers, migrated and invaded cell numbers per field. **p < 0.01, ***p < 0.001 versus NC-OE group, *n* = 3 [Student’s t-test]. *p < 0.05, **p < 0.01, ***p < 0.001 versus siNC group, *n* = 3 [one-way ANOVA followed by Dunnett’s post-hoc test]. **l** Macroscopic appearance of tumors from control and circUSP1-overexpressing group in subcutaneous xenograft mouse models. **m**, **n** Tumor growth curve and tumor weight of control and circUSP1-overexpressing group. *n* = 5. **o** HE staining of resected tumor tissues. The scale bar indicates 50 μm.

Next, we conducted gain- and loss-of-function experiments to assess the impact of circUSP1 on the progression of GC. We transfected MGC-803, AGS and SGC-7901 cells with circUSP1-overexpressing vector and two specific siRNAs targeting the back-spliced junction site of circUSP1 (Fig. 2a). Overexpression and knockdown efficiency of circUSP1 were verified without changing the USP1 mRNA level (Fig. 2b, c, Supplementary Fig. S2a, b). CCK8 assays showed that GC cell viability was significantly promoted with circUSP1 overexpression but suppressed with its knockdown compared to negative controls (Fig. 2d, e, Supplementary Fig. S2c). Colony formation assays also confirmed that circUSP1 overexpression significantly enhanced GC cell proliferation ability while its knockdown produced the opposite effects (Fig. 2f, g, Supplementary Fig. S2d, e). Furthermore, migration assays indicated that circUSP1 overexpression notably increased while its knockdown decreased the migration ability of GC cells (Fig. 2h, i, Supplementary Fig. S2f, g). Invasion assays presented that upregulated circUSP1 significantly enhanced cell invasion ability (Fig. 2j, Supplementary Fig. S2h). Conversely, downregulated circUSP1 suppressed GC cell invasion (Fig. 2k, Supplementary Fig. S2i). Additionally, the levels of proliferation-related protein PCNA and anti-apoptotic protein Bcl2 were upregulated while that of pro-apoptotic proteins Bax, caspase3 and caspase9 were downregulated after circUSP1 overexpression in MGC-803, AGS and SGC-7901 cells (Supplementary Fig. S2j). Mesenchymal markers N-cadherin and Vimentin levels were increased while epithelial marker E-cadherin levels were decreased when circUSP1 was upregulated (Supplementary Fig. S2j). CircUSP1 knockdown caused opposite results (Supplementary Fig. S2j).

Afterwards, we evaluated the effects of circUSP1 on GC progression in mouse models. In the subcutaneous xenograft mouse model, we found that the tumor size and growth speed in the circUSP1-overexpressing group were notably higher than that of the control group (Fig. 2l, m). Meanwhile, the circUSP1-overexpressing group exhibited significantly larger tumor weight (Fig. 2n). HE staining showed that all tumors were solid tumors (Fig. 2o). Taken together, these results suggest that circUSP1 exerts promotive effects on GC progression in vitro and in vivo.

### CircUSP1 interacts with HuR and inhibits its ubiquitination and degradation

**Fig. 3:**
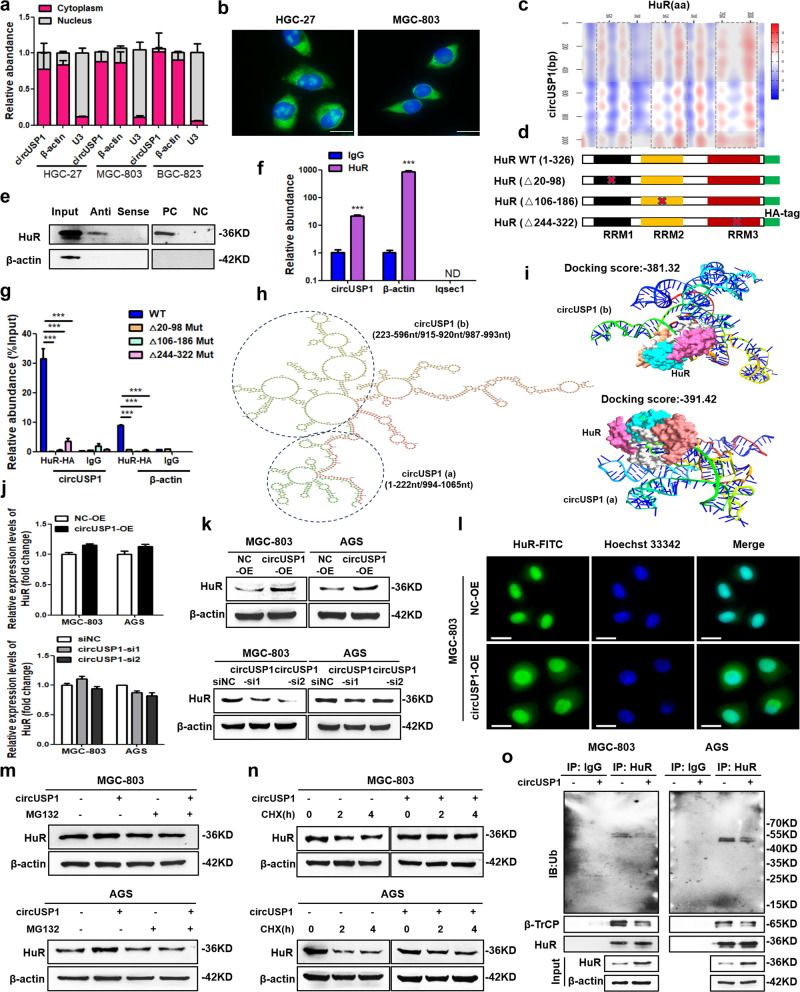
CircUSP1 interacts with HuR and inhibits its ubiquitination and degradation. **a** Cell fractionation analysis of circUSP1 in three GC cell lines. β-actin and U3 were used respectively as the positive control for cytoplasm and nucleus. **b** RNA FISH for circUSP1 in two GC cell lines using head-to-tail probes (labeled in green). Nuclei were counterstained with Hoechst 33342 (Blue). Scar bar = 10 μm. **c** Prediction of circUSP1-HuR interaction using the catRAPID algorithm. Potential interaction motifs of HuR and circUSP1 are marked in gray. **d** Schematic of HuR with functional RRMs. Wild-type HuR (HuR-WT) and three mutants lacking RRM1 (HuRΔ20-98), RRM2 (HuRΔ106-186) and RRM3 (HuRΔ244–322) motif are shown. **e** Biotin-labeled antisense (Anti) and sense probes were used for circUSP1-protein pull-down in MGC-803 cells. Positive control (PC) and negative control (NC) probes were applied for HuR. **f** RIP assay of circUSP1 in MGC-803 cells using anti-HuR antibodies. β-actin is the positive control for anti-HuR antibodies and Iqsec1 is the negative control. ND represents not detected. ***p < 0.001 versus lgG group, *n* = 3 [Student’s t-test]. **g** RIP analysis of circUSP1-overexpressing MGC-803 cells after transfected with HA-tagged WT and mutant HuR vectors using anti-HA antibodies and lgG control. β-actin is the positive control for anti-HuR antibodies. ***p < 0.001 versus WT group, *n* = 3 [one-way ANOVA followed by Dunnett’s post-hoc test]. **h** Secondary structure of circUSP1 predicted via RNAfold server. The potential interaction region of HuR was circled. **i** Graphical representation of the 3D structure of circUSP1 (region a and b) and HuR docking models using HDOCK. **j**, **k** The mRNA and protein levels of HuR after circUSP1 overexpression and knockdown. **l** Immunofluorescence analysis of HuR expression in cytoplasm and nucleus of MGC-803 cells with circUSP1 overexpression. Scar bar = 25 μm. **m** HuR protein level in circUSP1-overexpressing GC and control cells with or without MG132 (10 μM, 12 h) treatment. **n** HuR protein level in circUSP1-overexpressing and control GC cells treated with CHX (50 μg/ml) for 2 h or 4 h. **o** The ubiquitination of HuR and β-TrCP levels in GC cells with circUSP1 overexpression were determined by immunoprecipitation using anti-HuR antibodies followed by western blot using ubiquitin and β-TrCP antibodies.

To explore the underlying biological mechanism of circUSP1, its cellular distribution was initially examined. Cell fraction analyses presented that cytoplasmic circUSP1 level was much higher than that in the nucleus (Fig. 3a). RNA FISH results also showed that circUSP1 was primarily localized in the cytoplasm of GC cells (Fig. 3b, Supplementary Fig. S3a). It is commonly reported that cytoplasmic circRNAs often function as miRNA sponges. However, RIP assays revealed a relatively weak binding ability of circUSP1 with miRNAs-AGO2 complex (Supplementary Fig. S3b). Meanwhile, we examined circUSP1 in circRNADb (http://reprod.njmu.edu.cn/circrnadb) and found that it has a low potential to encode proteins due to the absence of an open reading frame (ORF). Then, we utilized the circInteractome algorithm (http://circinteractome.nia.nih.gov) to predict its interacting RBPs. FMRP, EIF4A3 and HuR were identified to have more binding sites with circUSP1 (Supplementary Fig. S3c). However, the catRAPID algorithm (http://service.tartaglialab.com/page/catrapid_group) indicated that HuR had much higher interaction propensity and lower discriminative power among them (Fig. 3c). To confirm these findings, we used biotin-labeled circUSP1 probes to specifically pull down circUSP1, and HuR was detected in circUSP1 precipitates (Fig. 3e). Additionally, we conducted RIP assays using the anti-HuR antibody which revealed that circUSP1 was present in the immunoprecipitates of HuR protein (Fig. 3f). These results suggest that HuR is capable of physically associating with circUSP1.

Based on the protein-RNA interaction heatmap, it appears that three RRMs (RRM1, RRM2 and RRM3) of HuR are likely the primary sites where circUSP1 binds (Fig. 3c). To verify this finding, we performed RIP assays using HA-tagged full-length and deletion mutants of HuR (Fig. 3d). The results showed that RRM3, RRM2 especially RRM1 was crucial for the interaction between HuR and circUSP1 (Fig. 3g). Additionally, we observed that the sequence segments spanning upstream 100nt and downstream 500 nt around the back-spliced junction might also serve as potential binding sites for HuR (Fig. 3c). To partly simulate the interaction between circUSP1 and HuR, we utilized RNAfold web server (http://rna.tbi.univie.ac.at/cgi-bin/RNAWebSuite/RNAfold.cgi) to predict the secondary structure of circUSP1. The minimum free energy (Δ*G* = 220.40 kcal/mol) was calculated (Fig. 3h). This secondary structure was then artificially divided based on the RNA stem-loop, among which regions a (1–222 nt/994–1065 nt) and b (223–596 nt/915–920 nt/987–993 nt) contained the potential binding sites. The primary sequence and secondary structure (Dot-Bracket Notation) of regions a and b were further inputted into RNAComposer (http:/rnacomposer.ibch.poznan.pl/) for tertiary structure, and HDOCK (http://hdock.phys.hust.edu.cn/) was applied for in silico molecular docking with the 3D structure of HuR modeled by SWISS-MODEL (https://swissmodel.expasy.org/) (Fig. 3i). The docking score was −391.42 and −381.32 for region a and b, respectively, supporting the interaction between circUSP1 and HuR.

Next, we investigated the potential role of circUSP1 in regulating the activity of HuR. Our results indicated that HuR mRNA level was not significantly changed in MGC-803, AGS and SGC-7901 cells with circUSP1-overexpressing and depleted (Fig. 3j, Supplementary Fig. S3d). However, the total level of HuR protein was increased with circUSP1 overexpression while decreased after circUSP1 knockdown (Fig. 3k, Supplementary Fig. S3e). Furthermore, circUSP1 overexpression led to a notable increase in the quantity of cytoplasmic HuR (Fig. 3l). These results suggested that circUSP1 can increase HuR protein at the post-transcriptional level. To confirm this hypothesis, we inhibited proteasome activity with MG132 and observed that the upregulation of endogenous HuR level caused by circUSP1 was prevented compared with the negative control (Fig. 3m). We also used cycloheximide (CHX) to inhibit protein synthesis and found that circUSP1 was able to prolong the half-life of the HuR protein (Fig. 3n). Moreover, Co-IP assays showed that circUSP1 overexpression markedly suppressed the interaction between HuR and E3 ubiquitin ligase β-TrCP and decreased the ubiquitous modification of HuR (Fig. 3o), suggesting that circUSP1 can prevent HuR degradation through the ubiquitin-proteasome pathway.

To further identify whether the upstream 100 nt to downstream 500 nt around the back-spliced junction is the key sequence of circUSP1 affecting HuR level, we constructed truncated a circUSP1 overexpressing vector (Supplementary Fig. S3f). Western blot results indicated that mutant circUSP1 did not change HuR protein level (Supplementary Fig. S3g). Moreover, CCK8 and colony formation assays showed that the promotive effects of circUSP1 in cell viability and proliferation ability were significantly reduced when mutated (Supplementary Fig. S3h, i). Its oncogenic effects on cell migration and invasion ability were also attenuated (Supplementary Fig. S3j, k). Additionally, we examined the effects of USP1 on HuR level since it shares the same exons with circUSP1. RT-qPCR and western blot results revealed that USP1 overexpression and knockdown did not affect HuR expression level (Supplementary Fig. S3l, m). These results suggested that the specific back-spliced sequence is crucial for circUSP1 oncogenic effects. Taken together, these findings revealed that circUSP1 plays an important role in stabilizing the HuR protein level by inhibiting its ubiquitination and degradation.

### HuR mediates the oncogenic effects of circUSP1 in GC progression

**Fig. 4:**
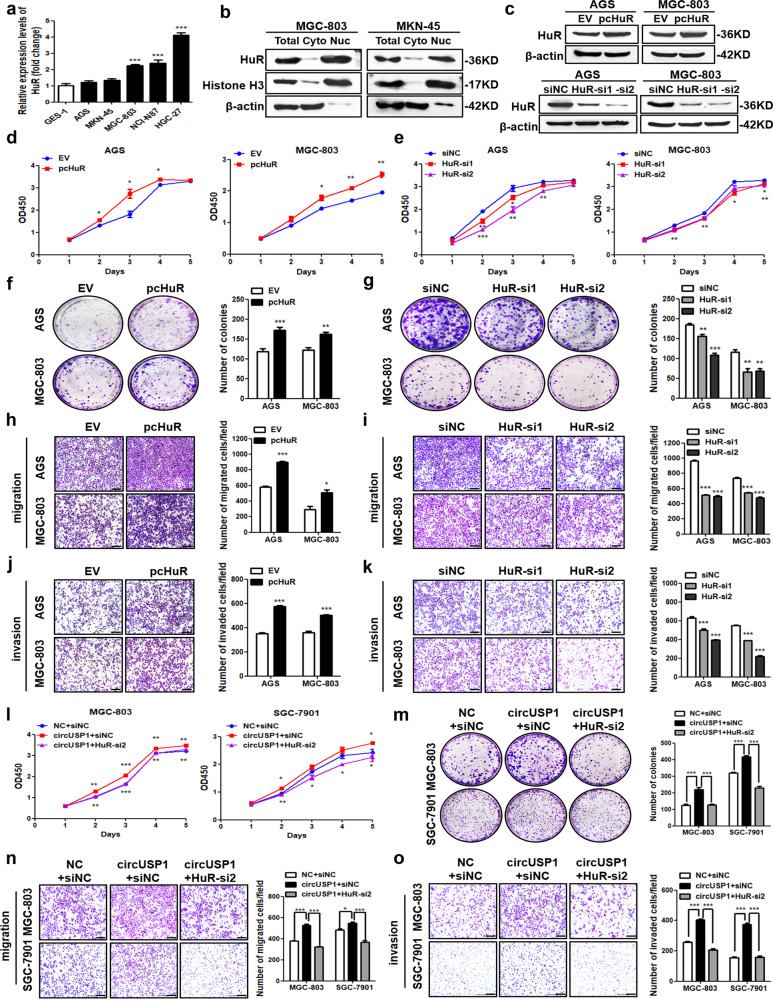
HuR mediates the oncogenic effects of circUSP1 in GC progression. **a** Comparison of HuR mRNA level in five GC cell lines and GES-1 cells with RT-qPCR analysis. ***p < 0.001 versus GES-1 group, *n* = 3 [one-way ANOVA followed by Dunnett’s post-hoc test]. **b** Cell fractionation analysis of HuR in GC cells with western blot. Histone H3 and β-actin were used respectively as the positive control for the nucleus (Nuc) and cytoplasm (Cyto). **c** Western blot analyses of HuR protein level in two GC cell lines after HuR overexpression and knockdown. CCK8 assays (**d**, **e**), colony formation assays (**f**, **g**), migration assays (**h**, **i**) and invasion assay (**j**, **k**) of GC cells with HuR overexpression and knockdown. The right bar graphs show the quantitative comparison of colony numbers, migrated and invaded cells per field. The scale bar indicates 200 μm. *p < 0.05, **p < 0.01, ***p < 0.001 versus EV group, *n* = 3 [Student’s t-test]. *p < 0.05, **p < 0.01, ***p < 0.001 versus siNC grou*p*, *n* = 3 [one-way ANOVA followed by Dunnett’s post-hoc test]. CCK8 (**l**), colony formation (**m**), migration (**n**) and invasion (**o**) assays of circUSP1-overexpressing GC cells co-transfected with HuR siRNAs. The right bar graphs show the quantitative comparison of colony numbers, migrated and invaded cells per field. The scale bar indicates 200 μm. *p < 0.05, **p < 0.01, ***p < 0.001 versus circUSP1+siNC group, *n* = 3 [one-way ANOVA followed by Bonferroni’s post-hoc test].

In our research, we observed that mRNA level of HuR was increased in most GC cell lines compared to normal GES-1 cells (Fig. 4a), and its protein was mainly located in the nucleus of GC cells (Fig. 4b). To confirm its biological roles in GC progression, HuR-overexpressing vector and two separate specific siRNAs were transfected into MGC-803, AGS and SGC-7901 cells to successfully upregulate and downregulate its expression in transcription (Supplementary Fig. S4a) and protein level (Fig. 4c, Supplementary Fig. S4b). CCK8 assays showed that GC cell viability was promoted with HuR overexpression but suppressed with its knockdown (Fig. 4d, e, Supplementary Fig. S4c). Colony formation assays confirmed that HuR overexpression increased while its knockdown decreased the proliferation ability of GC cells (Fig. 4f, g, Supplementary Fig. S4d, e). Moreover, migration assays presented that upregulated HuR enhanced while downregulated HuR inhibited GC cell migration ability (Fig. 4h, i, Supplementary Fig. S4f, g). Invasion assays indicated that HuR overexpression significantly enhanced cell invasion ability while its knockdown exhibited opposite effects (Fig. 4j, k, Supplementary Fig. S4h, i). These results suggested that HuR promotes GC proliferation, migration and invasion in vitro, which is consistent with circUSP1.

To confirm the crucial role of HuR in facilitating the effects of circUSP1 in GC progression, we performed rescue experiments in MGC-803 and SGC-7901 cells. CCK8 assays showed circUSP1 promoted GC cell viability, which was reversed by HuR knockdown efficiently (Fig. 4l). Similarly, colony formation assays revealed that the promoting effects of circUSP1 on GC cell proliferation could be suppressed by HuR knockdown (Fig. 4m). Furthermore, migration and invasion assays presented that the promoting effects of circUSP1 on GC cell metastasis could be markedly reversed when HuR was knocked down (Fig. 4n, o). Overall, these findings provided evidence that HuR plays a crucial role in mediating the oncogenic effects of circUSP1 on GC cell growth and metastasis.

### CircUSP1 increases USP1 and Vimentin expression via HuR-mediated post-transcriptional regulation

**Fig. 5:**
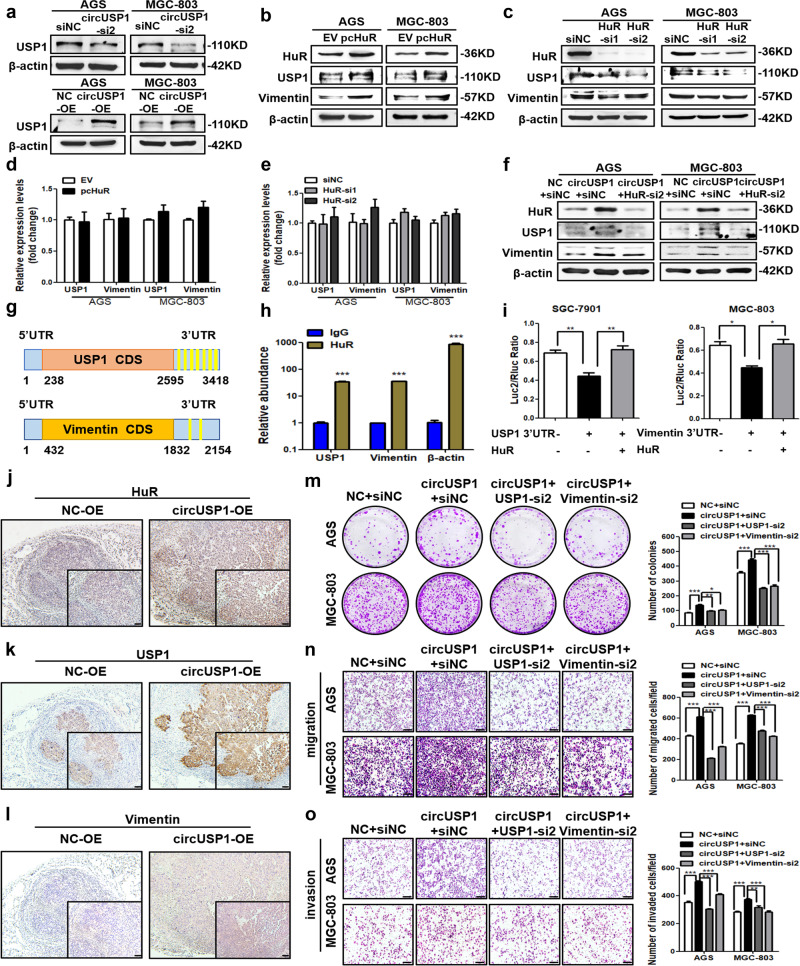
CircUSP1 increases USP1 and Vimentin expression via HuR-mediated post-transcriptional regulation. **a** The USP1 protein level in two GC cell lines with circUSP1 overexpression and knockdown. **b**–**e** The protein and mRNA levels of USP1 and Vimentin after HuR knockdown and overexpression in AGS and MGC-803 cells. **f** The protein levels of HuR, USP1 and Vimentin in circUSP1-overexpressing GC cells co-transfected with HuR siRNAs. **g** Schematic representation of AREs distribution (marked in bright yellow) in 3’UTR of USP1 and Vimentin mRNA. **h** RIP analysis of USP1 and Vimentin mRNA in circUSP1-overexpressing MGC-803 cells using anti-HuR antibodies. β-actin is the positive control gene for HuR. ***p < 0.001 versus lgG group, *n* = 3 [Student’s t-test]. **i** The regulatory effects of HuR on 3′ UTR in USP1 and Vimentin mRNA evaluated by dual-luciferase reporter gene assays. **p < 0.01 versus pmirGLO-USP1 3′ UTR + EV group, *n* = 3 [one-way ANOVA followed by Bonferroni’s post-hoc test]. *p < 0.05 versus pmirGLO-Vimentin 3′ UTR + EV group, *n* = 3 [one-way ANOVA followed by Bonferroni’s post-hoc test]. **j**–**l** IHC staining of HuR, USP1 and Vimentin in resected tumor tissues. The scale bar indicates 50 μm. Colony formation (**m**), migration (**n**) and invasion (**o**) assays of circUSP1-overexpressing GC cells co-transfected with USP1 and Vimentin siRNAs. The right bar graphs show the quantitative comparison of colony numbers, migrated and invaded cells per field. The scale bar indicates 200 μm. *p < 0.05, **p < 0.01, ***p < 0.001 versus circUSP1+siNC grou*p*, *n* = 3 [one-way ANOVA followed by Bonferroni’s post-hoc test].

We conducted further investigation into the targets influenced by circUSP1 through HuR. Since many circRNAs could affect the expression of their parent gene, it is still unknown if circUSP1 can regulate the USP1 level. We found that when circUSP1 was suppressed, the protein levels of USP1 together with Vimentin decreased markedly, while circUSP1 overexpression increased their levels (Fig. 5a, Supplementary Fig. S5a and 2j). However, the mRNA levels of USP1 and Vimentin remain unchanged (Fig. 2b, c, Supplementary Fig. S5b). Similarly, HuR positively regulated the protein levels of USP1 and Vimentin (Fig. 5b, c, Supplementary Fig. S5c) but their mRNA levels were not altered (Fig. 5d, e, Supplementary Fig. S5d). When we reduced the level of HuR in circUSP1-overexpressing GC cells, the upregulation of USP1 and Vimentin protein levels through circUSP1 was suppressed or reversed (Fig. 5f), suggesting that USP1 and Vimentin might be the downstream targets of HuR. Since HuR can bind to AU-rich elements (AREs) in the 3′ UTR of mRNAs and enhance their stability to promote gene translation [22], we hypothesized that HuR regulated the expression of USP1 and Vimentin at the post-transcriptional level. Sequence analysis showed that 3′ UTR of USP1 and Vimentin mRNA contained several copies of AREs including AUUUA pentamer or U-rich stretches (Fig. 5g, Supplementary SA1 and 2). Additionally, RIP assays demonstrated a strong binding ability between HuR and USP1 or Vimentin mRNA (Fig. 5h). To investigate the functional impact of these interactions, we inserted their 3′ UTR sequences into downstream of luciferase coding region of the pmirGLO vector (pmirGLO-USP1 3′ UTR/ pmirGLO-Vimentin 3′ UTR) and observed a decrease in the relative expression level of luciferase compared to the control (Fig. 5i, Supplementary Fig. S5e), which is consistent with previous reports that AREs mediate RNA instability when inserted to 3′ UTR of reporter genes [23]. However, when the pcHuR vector was co-transfected to upregulate HuR level, the relative expression of luciferase in pmirGLO-USP1 3’ UTR and pmirGLO-Vimentin 3′ UTR plasmids was increased significantly (Fig. 5i, Supplementary Fig. S5e). These results suggested that HuR can bind to 3′ UTR of USP1 and Vimentin mRNA and promote their expression. Taken together, our findings indicated that circUSP1 upregulates USP1 and Vimentin levels via HuR-mediated post-transcriptional regulation.

Furthermore, western blot and IHC results presented that the protein level of HuR was higher in tumors from the circUSP1-overexpressing group compared to the control group (Supplementary Fig. S5f, Fig. 5j), which was consistent with that observed in vitro (Fig. 3k). The protein levels of HuR target genes, USP1 and Vimentin, were also found to be upregulated simultaneously (Supplementary Fig. S5f, Fig. 5k, l). To verify whether USP1 and Vimentin are involved in the oncogenic effects of circUSP1, we reduced their expression in circUSP1-overexpressing GC cells (Supplementary Fig. S5g, h). CCK8 assays and colony formation assays showed that circUSP1 promoted GC cell viability and proliferation, which was suppressed by the knockdown of USP1 and Vimentin (Supplementary Fig. S5i, Fig. 5m). Similarly, migration and invasion assays revealed that the effects of circUSP1 promoting GC cell metastasis were markedly reversed when USP1 and Vimentin were knocked down (Fig. 5n, o). These findings demonstrated that USP1 and Vimentin, the target genes of HuR, mediate circUSP1 promotive effects on the growth and metastasis of GC cells.

### USP1 is upregulated in GC and promotes GC cell growth and metastasis

**Fig. 6:**
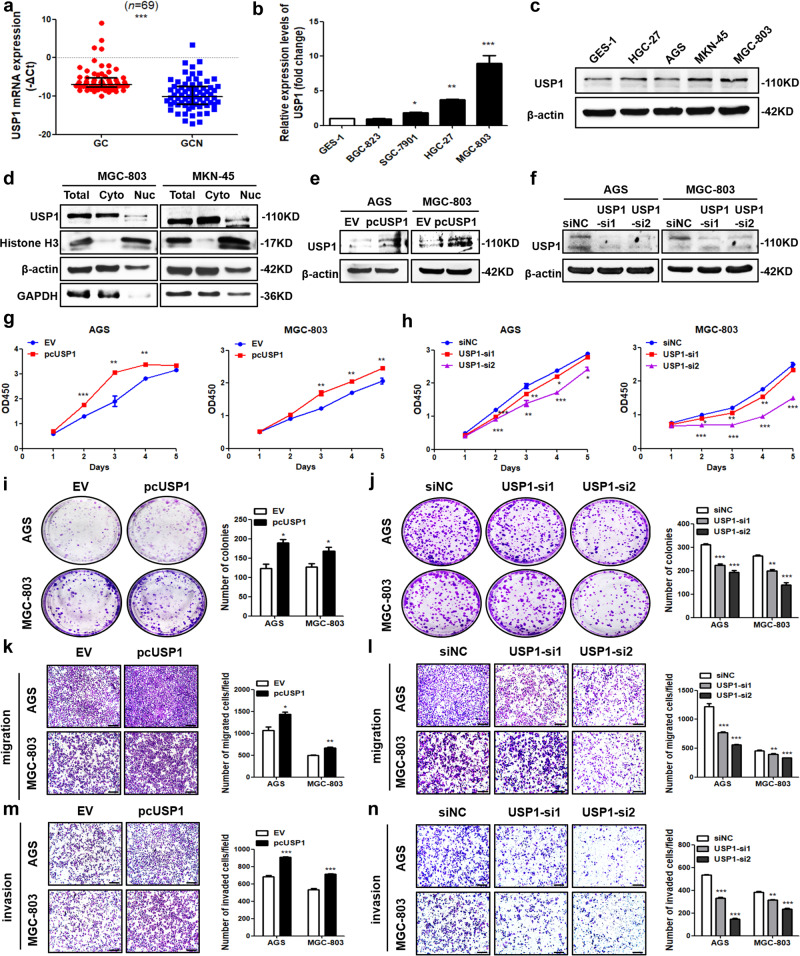
USP1 is upregulated in GC and promotes GC cell growth and metastasis. **a** RT-qPCR analysis of USP1 mRNA level in paired GC and adjacent normal (GCN) tissues. ***p < 0.001 versus GC group, *n* = 69 [Wilcoxon signed-rank test]. **b**, **c** RT-qPCR and western blot analyses of USP1 level in GES-1 and GC cells. *p < 0.05, **p < 0.01, ***p < 0.001 versus GES-1 group, *n* = 3 [one-way ANOVA followed by Dunnett’s post-hoc test]. **d** Cell fractionation analysis of USP1 expression in GC cells with western blot. β-actin and GAPDH are the positive control for cytoplasm (Cyto) while Histone H3 is a positive control for the nucleus (Nuc). **e**, **f** Western blot analyses for transfection efficiency of USP1 overexpression vectors and siRNAs in two GC cell lines. CCK8 assays (**g**, **h**), colony formation assays (**i**, **j**), migration (**k**, **l**) and invasion assays (**m**, **n**) after USP1 overexpression and knockdown. The bar graphs show the quantitative comparison of colony numbers, invaded and migrated cell numbers per field. The scale bar indicates 200 μm. *p < 0.05, **p < 0.01, ***p < 0.001 versus EV grou*p*, *n* = 3 [Student’s t-test]. *p < 0.05, **p < 0.01, ***p < 0.001 versus siNC group, *n* = 3 [one-way ANOVA followed by Dunnett’s post-hoc test].

RT-qPCR analysis showed that the level of USP1 mRNA was increased in GC tissues compared to adjacent normal tissues (Fig. 6a). Similarly, most GC cell lines had higher levels of USP1 mRNA and protein levels compared to normal GES-1 cells (Fig. 6b, c). Cell fractionation analysis showed that the USP1 protein was mainly located in the cytoplasm of GC cells (Fig. 6d). To verify the biological role of USP1 in GC progression, we transfected AGS, MGC-803 and SGC-7901 cells with USP1-overexpressing vector and two specific siRNAs to manipulate USP1 level. RT-qPCR and western blot analyses confirmed the transfection efficiency (Supplementary Fig. S6a, b, Fig. 6e, f). CCK8 and colony formation assays showed that the viability and proliferation ability of GC cells were enhanced with USP1 overexpression while suppressed after its knockdown (Fig. 6g–j, Supplementary Fig. S6c–e). Transwell assays presented that USP1 overexpression markedly promoted migration and invasion of GC cells (Fig. 6k, m, Supplementary Fig. S6f, h), while its knockdown exerts opposite effects (Fig. 6l, n, Supplementary Fig. S6g, i). Taken together, these results indicated that USP1 promotes the growth and metastasis of GC cells.

### CircUSP1 in peripheral blood is a potential diagnostic biomarker

**Fig. 7:**
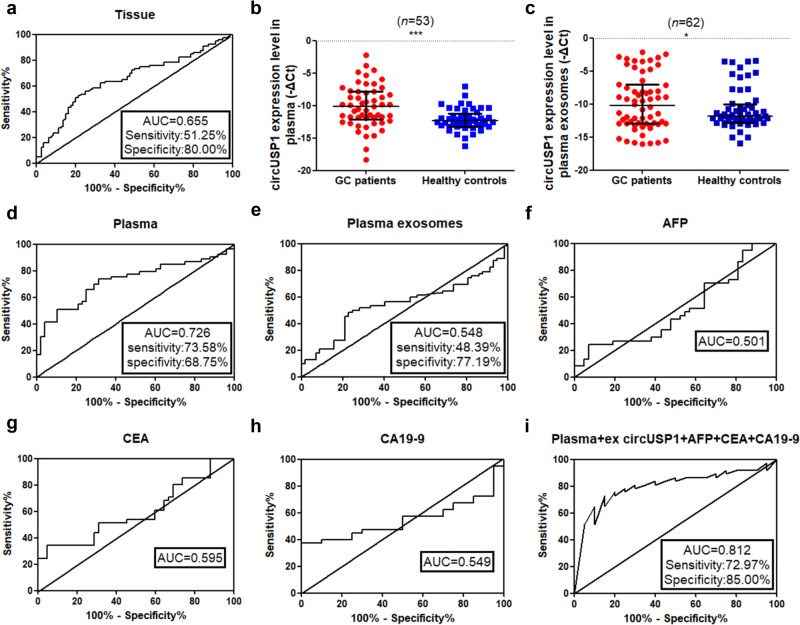
CircUSP1 in peripheral blood is a potential diagnostic biomarker. ROC curve analyses of tissue- (**a**), plasma- (**d**) and plasma exosome-derived (**e**) circUSP1 level and serum AFP (**f**), CEA (**g**) and CA19-9 (**h**) individually and in combination (**i**). Comparison of circUSP1 level in plasma (**b**) and plasma exosomes (**c**) from GC patients and healthy controls with RT-qPCR analysis. ***p < 0.001 versus GC patients group, *n* = 53 [Wilcoxon signed-rank test]. *p < 0.05 versus GC patients group, *n* = 62 [Wilcoxon signed-rank test].

We first investigated the clinical value of circUSP1 derived from tissue samples for GC diagnosis using ROC curve analysis. Our results showed that the area under the ROC curve (AUC) value was 0.655, suggesting a good diagnostic potential for GC. The sensitivity of the test was 51.25% and specificity was 80% under a cut-off value of −13.18 (Fig. 7a). Next, we measured the level of circUSP1 in plasma samples from preoperative GC patients and age- and gender-matched healthy donors. The results showed that GC patients had higher levels of circUSP1 in their plasma compared to the healthy controls (Fig. 7b). The AUC value for plasma-derived circUSP1 was 0.726, with a sensitivity of 73.58% and a specificity of 68.75% under the cut-off value of -11.85 (Fig. 7d), suggesting its good diagnostic value in distinguishing preoperative GC patients from healthy controls. Correlation analysis showed that high circUSP1 level in plasma was related to lymphatic metastasis (Table 3). The corresponding plasma exosomes were also isolated and tested. CircUSP1 level was still significantly upregulated in GC patients (Fig. 7c) which was associated with lymphatic metastasis and neural or vascular invasion (Table 4). The AUC value of plasma exosome-derived circUSP1 was 0.548, with a sensitivity of 48.39% and a specificity of 77.19% using the cut-off value of −9.748 (Fig. 7e). Overall, the diagnostic efficacy of plasma-derived circUSP1 was better than that of tissue- and plasma exosome-derived circUSP1.

**Table 3:** Relationship of circUSP1 expression levels (-△Ct) in plasma with clinicopathological factors of GC patients.

Characteristics	No.of cases	CircUSP1	
		Mean ± SD	*p* value
Age			
<60	10	−8.41 ± 3.52	0.067
≥60	43	−10.49 ± 3.08	
Gender			
Male	43	−10.16 ± 3.30	0.756
Female	10	−9.80 ± 3.11	
Tumor size			
<5 cm	32	−10.19 ± 2.90	0.785
≥5 cm	21	−9.94 ± 3.77	
Differentiation			
Poor	17	−10.68 ± 4.07	0.367
Moderate/Well	36	−9.82 ± 2.78	
Lymphatic metastasis			
N0	13	−9.91 ± 3.56	0.049*
N1	21	−9.82 ± 3.07	
N2	9	−12.61 ± 2.25	
N3	10	−8.63 ± 3.03	
Distal metastasis			
Absent	43	−10.26 ± 3.27	0.456
Present	10	−9.40 ± 3.15	
Neural invasion			
Absent	38	−10.22 ± 3.13	0.651
Present	15	−9.77 ± 3.59	
Vascular invasion			
Absent	46	−10.22 ± 3.80	0.456
Present	7	−9.24 ± 1.79	
TNM stage			
I	10	−10.56 ± 3.81	0.323
II	18	−9.20 ± 3.09	
III	15	−11.19 ± 2.82	
IV	10	−9.60 ± 3.38	
Invasion depth			
T1	5	−9.94 ± 2.95	0.898
T2	7	−10.61 ± 4.29	
T3	20	−9.69 ± 3.30	
T4	21	−10.34 ± 3.05	

Bold values indicate significance at *p* < 0.05.

**Table 4:** Relationship of circUSP1 expression levels (-ΔCt) in plasma exosomes with clinicopathological factors of GC patients.

Characteristics	No.of cases	CircUSP1	
		Median (IQR)	*p* value
Age (years)			
<60	12	−9.89 (−12.94∼−3.60)	0.373
≥60	50	−10.21 (−12.95∼−7.88)	
Gender			
Male	51	−9.28 (−12.89∼−6.98)	0.138
Female	11	−12.85 (−13.59∼−9.49)	
Tumor size (cm)			
<5	34	−11.63 (−12.91∼−8.12)	0.229
≥5	28	−8.40 (−13.28∼−4.16)	
Differentiation			
Poor	20	−9.95 (−12.54∼−4.20)	0.272
Moderate/Well	42	−10.40 (−13.11∼−7.86)	
Lymphatic metastasis			
N0	12	−12.76 (−14.92∼−8.41)	0.048*
N1	22	−11.07 (−13.36∼−8.30)	
N2	14	−10.46 (−13.14∼−3.43)	
N3	14	−8.16 (−10.68∼−3.39)	
Distal metastasis			
Absent	51	−10.80 (−12.97∼-6.98)	0.890
Present	11	−10.00 (−12.78∼-9.07)	
Neural/ Vascular invasion			
Absent	43	−11.48 (−13.06∼-8.15)	0.037*
Present	19	−8.12 (−12.12∼-3.97)	
TNM stage			
I	11	−12.85 (−13.59∼−7.55)	0.533
II	19	−8.97 (−12.97∼−8.11)	
III	22	−11.63 (−13.11∼−4.18)	
IV	10	−9.39 (−11.81∼−6.98)	
Invasion depth			
T1	4	−11.55 (−12.75∼−7.56)	0.330
T2	8	−12.90 (−15.01∼−4.47)	
T3	21	−9.28 (−13.92∼−8.25)	
T4	29	−9.54 (−12.38∼−4.02)	

Bold values indicate significance at *p* < 0.05.

In addition, we gathered and analyzed the levels of serum alpha-fetoprotein (AFP), carcinoembryonic antigen (CEA) and carbohydrate antigen 199 (CA19-9) levels from these GC patients and healthy controls. The AUC value was 0.501 for AFP, 0.595 for CEA and 0.549 for CA19-9 (Fig. 7f–h). However, when we combined plasma- or exosome-derived circUSP1 with these traditional tumor biomarkers, the AUC value significantly increased (Supplementary Fig. S7a). Among these combinations, the application of serum AFP, CEA, CA19-9, plasma- and exosome-derived circUSP1 together resulted in the highest AUC value of 0.812 (Fig. 7i), indicating the best diagnostic effectiveness. We also assessed the prognostic value of plasma-derived circUSP1. Kaplan-Meier survival analyses combined with log-rank tests showed that GC patients with higher expression of circUSP1 tended to have shorter post-operative survival time, although the statistical difference was not significant (Supplementary Fig. S7b). Therefore, circulating circUSP1 could serve as a new diagnostic biomarker for GC.

## Discussion

Tumor unlimited growth and metastasis are primary factors contributing to unfavorable prognosis and death of GC patients, as well as the biggest obstacles to clinical treatment. Thanks to the advancements in high-throughput RNA sequencing and bioinformatics, numerous circRNAs have been discovered in GC and are linked to disease progression [18]. Our study utilized microarray data and bioinformatics prediction to identify circUSP1 as a novel circRNA associated with GC. Moreover, our findings indicated that the circUSP1 molecule played a crucial role in the development and spread of GC. Clinicopathological characteristics analysis suggested that the level of circUSP1 in tumor tissues was associated with GC progression. Through laboratory experiments and animal models, we demonstrated that circUSP1 enhanced the proliferation, migration and invasion of GC cells. Previous studies have shown that tumor growth depends on the balance between the proliferation and apoptosis of tumor cells [24]. Additionally, the process of epithelial-mesenchymal transition (EMT) is important for the local invasion of primary tumor cells into surrounding tissues during tumor metastasis [25]. Our observations also indicated that circUSP1 stimulated the expression of proteins that promote cell proliferation and prevent apoptosis, as well as EMT-associated proteins. Therefore, circUSP1 likely promoted GC growth and metastasis by regulating processes such as proliferation, apoptosis and EMT.

Most of the cytoplasmic circRNAs have been identified as miRNA sponges to derepress downstream target mRNAs. However, we found that cytoplasmic circUSP1 interacted with HuR protein instead of miRNA-AGO2 complexes. Through bioinformatics analysis combined with RIP and RNA pull-down analyses, we confirmed the interaction between HuR and circUSP1. RIP assays and 3D molecular docking analysis further validated that the RRM3, RRM2 especially RRM1 motif was the key site for their interaction. More importantly, we discovered that circUSP1 upregulated cytoplasmic HuR at the protein level by suppressing its degradation. As a ubiquitous member of the Hu/elav RBPs family, HuR protein has been reported to be modulated by the ubiquitin-proteasome pathway where β-TrCP1 serves as a ubiquitin E3 ligase to recognize RRM3 of HuR for degradation [26]. Our further results verified that circUSP1 suppressed β-TrCP-mediated ubiquitination and degradation of HuR. Based on our findings, we proposed that circUSP1 might competitively inhibit the binding of β-TrCP to HuR. Through bioinformatic analysis and cell functional experiments based on truncated circUSP1, we also found that the sequence spanning back-spliced junction of circUSP1 was crucial for its effects on HuR level and oncogenic roles. Although USP1 mRNA shares the same exons with circUSP1, it cannot affect HuR expression level. Furthermore, it has been documented that HuR is increased in various types of cancers and contributes to tumor proliferation, invasion and metastasis by targeting multiple oncogenes, growth factors and cytokines [22]. The presence of elevated cytoplasmic HuR, rather than in the nucleus, is associated with the aggressive behavior of and poor prognosis of tumors [27]. In this study, we observed that HuR protein was accumulated in the cytoplasm of GC cells stimulated by circUSP1. Our cell functional experiments confirmed the promotive effects of HuR on GC cell growth and metastasis. Additionally, rescue functional experiments provided further evidence for the crucial role of HuR in mediating the oncogenic effects of circUSP1 in GC progression. Taken together, HuR served as an important mediator for the promotive role of circUSP1 in the progression of GC.

Ubiquitin-specific protease 1 (USP1) is a type of enzyme known as deubiquitinases (DUBs) which is involved in maintaining cell homeostasis by reversing protein ubiquitination [28]. In various types of cancer, USP1 is often overexpressed and mediates the stabilization of inhibitors of DNA binding and cell differentiation (ID proteins family) to regulate tumor proliferation, metastasis and apoptosis [29]. For example, it deubiquitinates and stabilizes ID1, ID2 and ID3 proteins to enhance the proliferation of osteosarcoma [30]. In GC, USP1 has been recently reported to promote tumor metastasis by stabilizing ID2 expression [31]. Here, we found that USP1 was upregulated in GC tissues and cells, and it promoted GC cell proliferation, migration and invasion. More importantly, we discovered the novel mechanisms for USP1 dysregulation in GC that circUSP1 stabilized HuR to enhance the post-transcriptional upregulation of USP1. Both circUSP1 and HuR upregulated USP1 protein expression without affecting its transcription level in GC cells. Our sequence analysis and molecular experiments further proved that HuR was able to bind to AREs of USP1 in 3′ UTR and enhanced its expression. When HuR was knocked down in circUSP1-overexpressing cells, the USP1 level markedly decreased. These results suggested that USP1 was a new downstream target of HuR and a critical post-transcriptional regulator that stabilized specific mRNAs by competing with decay factors in 3′ UTR [23]. Some circRNAs have been reported to regulate their parental gene transcription by interacting with transcription factors or promoters in the nucleus [17, 32, 33]. In this study, we found a circRNA circUSP1 regulating its host gene expression by increasing the stability of its mRNA in the cytoplasm. In addition, we also identified Vimentin as a new gene targeted by HuR. It is a well-known marker of EMT and related to the increased migratory or invasive capacity of cancer cells [34]. Hence, circUSP1 contributed to the progression of GC by increasing the levels of USP1 and Vimentin through the actions of HuR.

Early diagnosis and accurate evaluation of the prognosis are crucial in improving the outlook for GC patients. However, traditional tumor biomarkers, such as serum AFP, CEA and CA19-9, have limited diagnostic capacity, sensitivity and specificity [35–37]. Using the TNM stage to predict the OS of GC patients also has its limitations in terms of accuracy and specificity. Fortunately, there is promising potential to apply novel circRNA biomarkers. These circRNAs have unique properties such as high stability, conservation and widespread distribution in human body fluids, especially in exosomes, which makes them easily detectable using non-invasive methods [38]. Additionally, circRNAs have tissue/developmental-stage-specific expression pattern, which allows them to reflect the pathological process of tumors. Some specific circRNAs, like hsa_circ_0000096 derived from tissues and hsa_circ_0130810 derived from plasma, have demonstrated good diagnostic efficacy for GC with the AUC value of 0.82 and 0.748 [39, 40]. Furthermore, circPVT1 and circOSBPL10 have been identified as prognostic markers that are closely associated with the OS of GC patients [20, 41]. In this study, circUSP1 was able to serve as a new diagnostic and prognostic biomarker for GC. We conducted RNase R and half-life assays to prove its higher stability compared to the linear transcript. Our ROC curve analysis confirmed that circUSP1 derived from plasma had better diagnostic value compared to circUSP1 derived from tissue and plasma exosomes, as well as serum AFP, CEA and CA19-9. The combination of plasma- and plasma exosome-derived circUSP1, along with serum AFP, CEA and CA19-9, improved the diagnostic efficiency and might offer a new strategy for GC diagnosis in the future. Additionally, our Cox regression and Kaplan-Meier survival analyses verified that upregulated tissue-derived circUSP1 was an independent poor prognostic marker in GC. However, more investigation is needed to fully understand the role of circUSP1 in diagnosing and predicting the prognosis of GC.

Molecular targeted therapy using small molecular inhibitors and monoclonal antibodies targeting HER2 or VEGF has shown improved anti-cancer efficacy, especially in advanced and metastatic GC compared with traditional surgical resection, radiation and chemotherapy [42]. However, the obstacles including drug resistance and limited druggable targets remain to be overcome [43, 44]. CircRNAs have shown great potential as new druggable targets due to their significant regulatory roles in tumor progression. RNA interference (RNAi) technology, utilizing siRNAs or short hairpin RNAs (shRNAs), might be used to disrupt oncogenic circRNAs [45]. In our study, specific siRNAs were used to decrease the level of oncogenic circUSP1, resulting in significant suppression of GC growth and metastasis in vitro. Besides, nano-based drug delivery systems can be applied to improve the biostability, permeability and tumor targetability of RNA-based drugs [46]. However, the real anti-cancer efficacy and biological safety remain to be validated in the future.

**Fig. 8:**
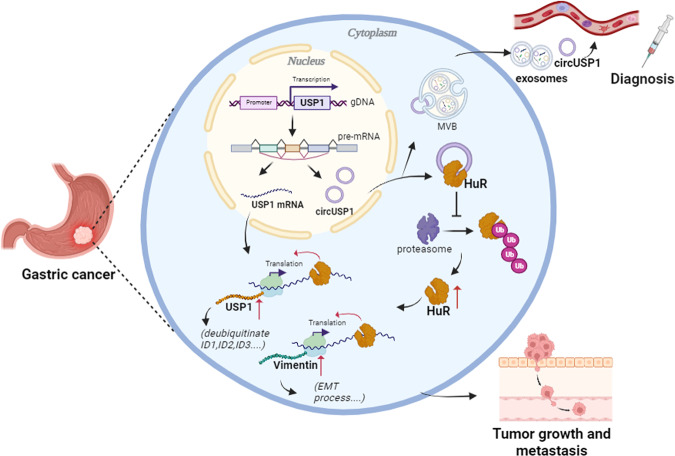
Proposed model for the mechanism of action and application of circUSP1 in GC. CircUSP1 serves as a novel diagnostic biomarker for GC and promotes GC growth and metastasis by interacting with and stabilizing oncogenic HuR to enhance its post-transcriptional upregulation of USP1 and Vimentin.

In conclusion, we have identified circUSP1, a new GC-associated circRNA, that is increased in GC tissues and circulating blood and correlates with clinicopathological factors and poor prognosis. Additionally, we have confirmed that circUSP1 promotes GC growth and metastasis by interacting with and stabilizing oncogenic HuR to enhance its post-transcriptional upregulation of USP1 and Vimentin (Fig. 8). Our findings uncover a new mechanism for GC progression and offer a novel potential target for future GC diagnosis and therapy.

## Materials and methods

### Collection of clinical samples and patient data

Paired GC tissues and adjacent normal tissues were obtained from GC patients who received surgical resection at the Affiliated Tumor Hospital of Nantong University from 2018 to 2019 (*n* = 50) and the Affiliated People’s Hospital of Jiangsu University from April 2015 to September 2016 (*n* = 69). Paired plasma samples (*n* = 62) from preoperative GC patients and matched healthy donors in terms of age and gender were obtained at the Department of Clinical Laboratory, the Affiliated People’s Hospital of Jiangsu University from January 2017 to July 2018 (Supplementary Fig. S1c). The inclusion criteria were patients who had a confirmed pathological GC diagnosis with complete pathological data. Patients who had other forms of cancer or received chemotherapy or radiotherapy before surgery were excluded. The tissues were frozen at −80 °C until use. The intravenous blood samples (3 mL) were collected in EDTA-K2 anti-coagulant tubes and then centrifugated at 1000 × *g* for 10 min at 4 °C following a second centrifugation at 3000 × *g* for 15 min at 4 °C to isolate plasma. Each plasma sample was aliquoted and stored at −80 °C for further use. The pathology reports and clinical data were obtained from hospital medical records. Patients were followed up and the time interval was from the date of surgery to the date of clinically defined recurrence, disease progression or death. The Institutional Ethical Committee of Jiangsu University approved this study (IRB protocol number: 2020161) and written informed consent was obtained from all participants.

### Plasma exosome isolation

Exosomes from human plasma were isolated using ExoQuick exosome precipitation solution (SBI, Mountain View, CA, USA). Each volume of 250 μL plasma was mixed with 63 μL ExoQuick solutions and incubated at 4 °C overnight. Exosome pellets were collected after centrifugation at 1500 × *g* for 30 min and then suspended in 50 μL sterile PBS. All exosomes were aliquoted and stored at −80 °C until use.

### Cell lines and culture conditions

Human gastric epithelial cell line GES-1 was purchased from Gefan Biological Technology (Shanghai, China). Human GC cell lines MGC-803, AGS, SGC-7901 and HGC-27 were bought from the Cell Bank of the Chinese Academy of Sciences (Shanghai, China). All cell lines were regularly tested for Mycoplasma. MGC-803 cells were maintained in high-glucose DMEM (Gibco, USA). AGS cells were cultured in DMEM/F-12 medium (Bioind, Israel). SGC-7901 and HGC-27 cells were propagated in RPMI-1640 (Bioind, Israel). The cell-culture medium was supplemented with 10% fetal bovine serum (FBS; Bovogen, Australia) before use. All cell lines were cultured at 37 °C with a 5% CO_2_ atmosphere following the standard protocol.

### RNA extraction and reverse transcription-quantitative polymerase chain reaction (RT-qPCR)

Total RNA was isolated from tissues and cell lines using TRIzol reagent (Invitrogen, Carlsbad, CA, USA). Total RNA from plasma and plasma exosomes was isolated and purified using miRNeasy Serum/Plasma kit (Qiagen, Germany). RNA concentration and purity were measured by NanoDrop 2000 (Thermo Fisher Scientific, USA). RNA was reverse transcribed into cDNA with HiScript 1st Strand cDNA Synthesis Kit (Vazyme, Nanjing, China) and then quantified by quantitative PCR with AceQ qPCR SYBR Green Master Mix (Vazyme, Nanjing, China). RT-qPCR analysis was conducted on Bio-Rad CFX96 Real-Time PCR system (Bio-Rad, USA) and β-actin was used as an internal control. RT-qPCR products were separated by 1.5% agarose gels, examined by UV irradiation and verified by Sanger sequencing. The expression level was presented by the -△Ct method and the relative expression level was calculated by the 2^-△△Ct^ method. Primer sequences for circRNAs and mRNAs were listed in Supplementary Table S1.

### Cell nuclear and cytoplasmic fractionation, RNase R and actinomycin D treatment

Cytoplasmic RNA was isolated with RNeasy Mini Kit (Qiagen, Germany) and the remaining nuclear RNA was extracted with TRIzol reagent (Invitrogen, Carlsbad, CA, USA). Nuclear and cytoplasmic protein fractionation was performed using the Nuclear and Cytoplasmic Extraction Kit (Vazyme, Nanjing, China). For RNase R treatment, 2 μg total RNA was incubated with 6 U RNase R enzyme in 1× buffer (Epicentre, USA) for 30 min at 37 °C. RNA was purified using the ethanol precipitation method according to the standard protocol. For actinomycin D treatment, MGC-803 cells (2 × 10^5^/well) were seeded in 6-well plates overnight and treated with 2 mg/mL actinomycin D or DMSO control (Sigma-Aldrich, USA) for 0 h, 4 h, 8 h, 12 h, 18 h and 24 h. Total RNA was then isolated for RT-qPCR analysis.

### RNA fluorescence in situ hybridization (FISH)

RNA FISH for circUSP1 was performed using the FISH kit (GenePharma, Shanghai, China). Cells (1×10^4^/well) were grown on round coverslips in 24-well plates overnight. After fixed with 100% ethanol, permeabilized with 0.1% triton X-100, incubated with 2× saline sodium citrate (SCC) and dehydrated in ethanol, cells were hybridized with fluorescence-labeled RNA probes at 37 °C overnight and counterstained with Hoechst 33342 (Sigma, USA) for 10 min at room temperature. Laser scanning confocal microscopy (Nikon, Japan) was used to visualize and obtain images. The probe sequence for circUSP1 was as follows: 5′- CAUACAUAGGGUUGAGUUCCUCCAAACCACUAGCAGCAA-3′.

### Transfection, oligonucleotides and plasmids

Two separate small interfering RNAs (siRNAs) targeting HuR, USP1, Vimentin and the back-spliced junction of circUSP1 were synthesized respectively for gene knockdown (GenePharma, Shanghai, China). A nonspecific siRNA was used as the negative control (GenePharma, Shanghai, China). The siRNAs sequences were listed in Supplementary Table S2. CircUSP1 overexpression vector (circUSP1-OE) was constructed via synthesizing circUSP1 sequence flanking circularization elements and inserting it into pcDNA3.1(+) vector (GenePharma, Shanghai, China) and its empty vector was set as a negative control (NC-OE). CircUSP1 mutant vector (circUSP1-Mut) was constructed by inserting truncated circUSP1 sequence flanking circularization elements into pcDNA3.1(+) vector (GenScript, Nanjing, China). Overexpression plasmid vectors for USP1 (pcUSP1) and HuR (pcHuR) were constructed by inserting their coding sequence (CDS) region into pcDNA3.1(+) vector (GenePharma, Shanghai, China) while empty vector (EV) was used as the negative control. HA-tagged wild-type (WT) and three RNA-recognition motifs (RRMs) deletion mutants of HuR vectors were constructed by GenScript (Nanjing, China). GC cells seeded in 6-well plates (2 × 10^5^/well) or 10-cm dishes (8 × 10^5^/dish) overnight were transfected with the above siRNAs (25 nM) or plasmids (500 ng) using Lipofectamine 2000 (Invitrogen, Carlsbad, CA, USA) in serum-free medium for 6 h and changed with complete medium. Cells were harvested after 48 h for RNA analysis or functional experiments and 72 h for protein analysis.

### Dual-luciferase reporter gene assay

Recombinant dual-luciferase reporter plasmids (pmirGLO-USP1 3′ UTR and pmirGLO-Vimentin 3′ UTR) were synthesized by GenScript (Nanjing, China) via inserting 3′ untranslated region (3′ UTR) sequences of USP1 and Vimentin into pmirGLO dual-luciferase expression vector (Promega, USA). MGC-803 cells (4 × 10^4^/well) were seeded in 24-well plates overnight before 400 ng luciferase reporter vector were co-transfected into cells with 400 ng pcHuR or EV control. After 48 h, the cells were harvested and analyzed for firefly and Renilla luciferase activity using Dual-Glo Luciferase Assay Kit (Promega, USA) on GloMax 20/20 Luminometer (Promega, USA). Firefly luciferase activity was normalized to Renilla luciferase activity.

### RNA immunoprecipitation (RIP) assay

RIP assays were conducted using the Magna RIP RNA-Binding Protein Immunoprecipitation Kit (Millipore, Billerica, USA). Briefly, MGC-803 cells were lysed in RNA lysis buffer with protease and RNase inhibitors. The cell lysates were incubated with magnetic beads conjugated with 5 μg negative control lgG (Cat# CS200621, Cat# PP64B, Millipore, Billerica, USA), positive control anti-snRNP70 antibody (Cat# CS203216, Millipore, Billerica, USA), mouse anti-AGO2 antibody (Cat# 03-110, RRID: AB_10615426, Millipore, Billerica, USA), rabbit anti-HuR antibody (Cat# 03-102, RRID: AB_11211202, Millipore, Billerica, USA) or Mouse anti-HA antibody (Cat# AE008, RRID: AB_2770404, ABclonal, USA) and rotated at 4 °C overnight. The beads were then washed and digested with proteinase K buffer at 55 °C for 30 min to remove proteins. The immunoprecipitated RNA was extracted and purified for RT-qPCR analysis.

### Biotin-labeled RNA pull-down assay

RNA pull-down assays were performed using the Pierce Magnetic RNA-Protein Pull-Down Kit (Thermo Fisher Scientific, USA). Biotinylated sense and antisense probes for circUSP1 were synthesized by GenePharma (Shanghai, China). Positive and negative RNA probes contained in the kit were set as controls to pull down HuR protein. Briefly, 50 pmol biotinylated probes were labeled to 50 μL streptavidin magnetic beads and then mixed with MGC-803 cell lysates at 4 °C for 1 h. After washing and elution, the RNA-binding protein complexes were subjected to western blot analysis for HuR. The probe sequences were as follows: 5′- CAUACAUAGGGUUGAGUUCCUCCAAACCACUAGCAGCAA-3′ biotin (antisense), 5′- UUGCUGCUAGUGGUUUGGAGGAACUCAACCCUAUGUAUG-3′ biotin (sense).

### Co-immunoprecipitation (Co-IP) assay

CircUSP1-overexpressing MGC-803, AGS and their control cells were extracted in 1 mL IP lysis Buffer (Beyotime, China) and incubated on ice for 10 min, followed by sonication and centrifugation at 12,000 × *g* for 10 min at 4 °C. Each volume of 150 μL clarified supernatant was incubated with 5 μL anti-HuR (Cat# A19622, RRID: AB_2862702, ABclonal, USA) and negative control lgG antibodies (Cat# PP64B, Millipore, Billerica, USA) at 4 °C overnight and then added pre-washed protein A/G-agarose beads (Beyotime, China) for 3 h at 4 °C. After washing with IP lysis Buffer and centrifugation at 3000 × *g* for 5 min at 4 °C for three times, proteins were eluted at 95 °C for 10 min subjected to western blot analysis for ubiquitin and β-TrCP.

### CCK8 and colony formation assays

For CCK8 assays, 100 μL complete medium containing GC cells (4 × 10^3^) were maintained in triplicate 96-well plates for 1–5 days respectively. Then, 10 μL CCK-8 solution (Vazyme, Nanjing, China) was added to each well and incubated at 37 °C for 2 h. The absorbance at 450 nm was measured with the microplate reader (BioTEK, USA) to evaluate cell viability. For colony formation assays, GC cells (2 × 10^3^/well) were cultured in 6-well plates and the culture medium was changed every 2–3 days. After 10–14 days, colonies were fixed with 4% paraformaldehyde and stained with 0.1% crystal violet. The colonies were imaged and accurately counted.

### Migration and invasion assays

Migration and invasion assays were performed by the Transwell system (Corning, USA). The lower chamber was added with 600 μL complete medium as a chemoattractant. GC cells (1 × 10^5^) in 200 μL serum-free medium were seeded into the upper chamber with (invasion assay) or without (migration assay) Matrigel (BD Bioscience, USA) coating. After incubating at 37 °C for 12 h (migration assay) or 24 h (invasion assay), the upper chambers were fixed in 4% paraformaldehyde and stained with 0.1% crystal violet. Cells remaining at the upper surface of the membrane were removed with cotton swabs while migrated or invaded to lower surface cells were photographed and counted under a microscope (Olympus, Japan).

### Immunofluorescence (IF)

GC cells (1 × 10^4^) were cultured on coverslips overnight and fixed with 4% paraformaldehyde at room temperature for 30 min. Then, cells were permeabilized with 0.1% Triton-100 and blocked with 5% bovine serum albumin (BSA) for 30 min at room temperature before being incubated with rabbit anti-HuR primary antibodies (Cat# 03-102, RRID: AB_11211202, 1:200, Millipore, USA) overnight at 4 °C and secondary FITC goat anti-rabbit lgG antibodies (Cat# AS011, RRID: AB_2769476, 1:300, ABclonal, USA) at 37 °C for 1 h. After counterstaining with Hoechst 33342 (1:500, Sigma, USA), cells were photographed in laser scanning confocal microscopy (Nikon, Japan).

### Western blot

Total proteins from GC cells were harvested with RIPA lysis buffer (Pierce, USA) containing proteinase inhibitors on ice. Equal amounts of proteins were loaded and separated with SDS-polyacrylamide gel electrophoresis (SDS-PAGE). Then, proteins were transferred onto the polyvinylidene fluoride (PVDF) membrane (Millipore, USA), blocked in 5% (W/V) non-fat milk and incubated with primary antibodies at 4 °C overnight. The primary antibodies were as follows: rabbit anti-β-actin antibody (Cat# AC026, RRID: AB_2768234, 1:5000, ABclonal, USA), rabbit anti-Bax antibody (Cat# 2772, RRID: AB_10695870, 1:1000, Cell Signaling, USA), mouse anti-Bcl2 antibody (Cat# 15071, RRID: AB_2744528, 1:200, Cell Signaling, USA), rabbit anti-actived-Caspase-3 antibody (Cat# BS7004, RRID: AB_1662741, 1:500, Bioworld, USA), mouse anti-Caspase-9 antibody (Cat# 9508, RRID: AB_2068620, 1:600, Cell Signaling, USA), rabbit anti-PCNA antibody (Cat# BS1289, RRID: AB_1663403, 1:800, Bioworld, USA), rabbit anti-E-cadherin antibody (Cat# sc-7870, RRID: AB_2076666, 1:300, Santa Cruz, USA), rabbit anti-N-Cadherin antibody (Cat# 13116, RRID: AB_2687616, 1:300, Cell Signaling, USA), rabbit anti-Vimentin antibody (Cat# BS1491, RRID: AB_1663663, 1:800, Bioworld, USA), rabbit anti-USP1 antibody (Cat# 8033, RRID: AB_10858879, 1:250, Cell Signaling, USA), rabbit anti-HuR antibody (Cat# 03-102, RRID: AB_11211202, 1:1000, Millipore, Billerica, USA), rabbit anti-GAPDH (Cat# AC001, RRID: AB_2619673, 1:5000, ABclonal, USA), rabbit anti-Histone H3 (Cat# 9733, RRID: AB_2616029, 1:2000, Cell Signaling, USA), mouse anti-ubiquitin (Cat# 3936, RRID: AB_331292, 1:500, Cell Signaling, USA), rabbit anti-β-TrCP (Cat# A1656, RRID: AB_2763713, 1:1500, ABclonal, USA). After washing by tris-buffered saline with tween 20 (TBS-T) three times, the membrane was incubated with goat anti-rabbit lgG secondary antibody (Cat# 31460, RRID: AB_228341, 1:2000, Invitrogen, USA) or goat anti-mouse lgG secondary antibody (Cat# 31430, RRID: AB_228307, 1:1000, Invitrogen, USA) at 37 °C for 1 h. Each protein band was visualized by the enhanced chemiluminescence system (ImageQuant LAS4000mini, GE, Japan).

### Subcutaneous xenograft tumor model

Male BALB/c nu/nu mice (Cavens, Changzhou, China) aged 4–6 weeks were randomly divided into two groups (*n* = 5) and received the subcutaneous injection of either circUSP1-overexpressing MGC-803 cells or negative control cells (4 × 10^6^ cells in 200 μL PBS per mouse). After 2 weeks, all mice were sacrificed and the subcutaneous tumors were harvested for protein analysis or fixed with 4% paraformaldehyde and made into paraffin-embedded tissue sections. Tumor formation data were calculated for blinded groups, which were then unblinded by the investigators. The animal experiment was approved by the Institutional Animal Care and Use Committee of Jiangsu University (2012258) and performed following the guidelines for the care and use of laboratory animals.

### Hematoxylin-eosin (HE) staining and immunohistochemistry (IHC)

For HE staining, paraffin-embedded tissue sections (5 mm thick) were dewaxed in xylene, rehydrated through decreasing concentrations of ethanol, washed in PBS and stained with HE. For IHC staining, the SABC kit was used (Boster, China). Sections were deparaffinized in xylene, rehydrated through graded ethanol and boiled in citrate buffer (10 mM, PH 6.0) for 30 min to retrieve antigen. After exposure to 3% hydrogenous peroxidase for 10 min to suppress endogenous peroxidase activity, sections were blocked in 5% BSA, incubated with primary antibody at 4 °C overnight, secondary antibody at 37 °C for 30 min and streptavidin-biotin complex (SABC) at 37 °C for 30 min. The tissue sections were finally visualized with diaminobenzidine (DAB) and counterstained with hematoxylin for microscopic observation. After being mounted in a neutral resin medium, sections were scanned with the digital slide scanner Pannoramic MIDI (3DHistech, Hungary). The primary antibodies were as follows: rabbit anti-HuR antibody (Cat# 03-102, RRID: AB_11211202, 1:100, Millipore, Billerica, USA), rabbit anti-USP1 antibody (Cat# 8033, RRID: AB_10858879, 1:100, Cell Signaling, USA), rabbit anti-Vimentin antibody (Cat# BS1491, RRID: AB_1663663, 1:100, Bioworld, USA).

### Statistical analysis

Statistical analysis was performed with SPSS 21.0 (IBM, Chicago, IL, USA) and GraphPad Prism version 5.0 software (LaJolla, CA, USA). All experiments were repeated at least three times. Data are presented as median (IQR) or mean ± SD where appropriate. Shapiro-Wilk test was used to evaluate the normality of data distribution and Levene’s test was performed to verify the homogeneity of variance. A two-tailed Student’s t-test or Wilcoxon signed-rank test was used to compare paired continuous variables. One-way ANOVA or Kruskal Wallis test was for the comparison of three or more groups. Univariate and multivariate Cox regression analyses were applied to evaluate different prognostic variables. Overall survival analysis was conducted by log-rank tests in Kaplan-Meier plots. For all results, *p* < 0.05 were considered statistically significant.

### Supplementary information

Supplementary results

Supplementary tables

## Data Availability

The data used to support the findings of this study are available from the corresponding author on reasonable request.
