# Supplementary material for: CircUSP1 as a novel marker promotes gastric cancer progression via stabilizing HuR to upregulate USP1 and Vimentin
Source: Oncogene. 2024 Feb 16;43(14):1033–49. doi: 10.1038/s41388-024-02968-8 (PMC10978489; doi:10.1038/s41388-024-02968-8)
Supplement: Supplementary file 1 — Supplementary results [file 41388_2024_2968_MOESM1_ESM.docx]

**Supplementary results**


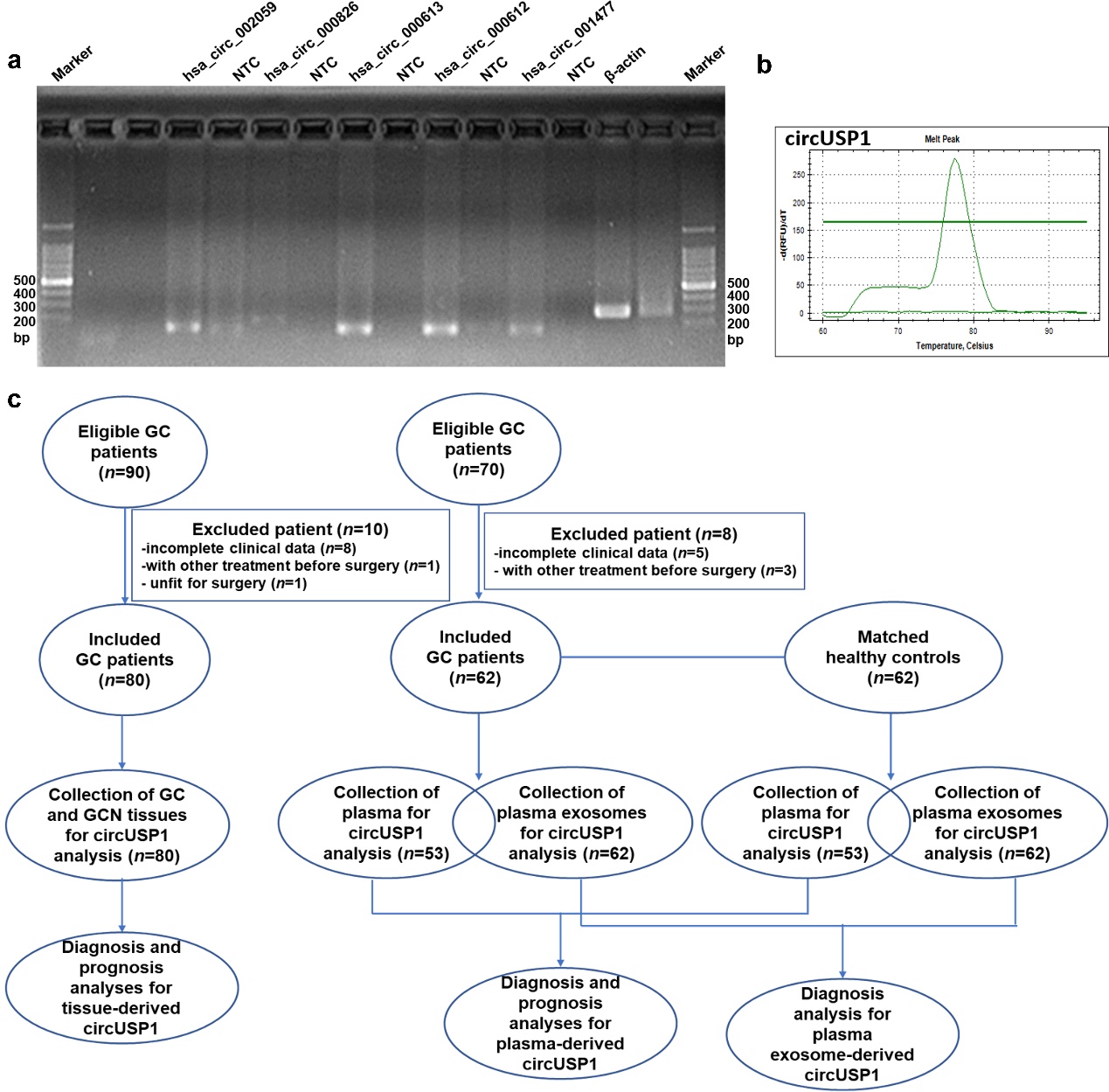


**Fig.S1 RT-qPCR analysis of GC-related circRNAs.** **a** Agarose gel electrophoresis analysis for RT-qPCR products of hsa_circ_002059 (126bp), hsa_circ_000826 (184bp), hsa_circ_000613 (120bp), hsa_circ_000612 (120bp) and hsa_circ_001477 (119bp) from MGC-803 cells. NTC represents negative control. **b** Melt curve of RT-qPCR analysis for hsa_circ_000613 (circUSP1). **c** Flowchart illustrating the patient selection for diagnosis and prognosis analyses of tissue-, plasma-, and plasma exosome-derived circUSP1.


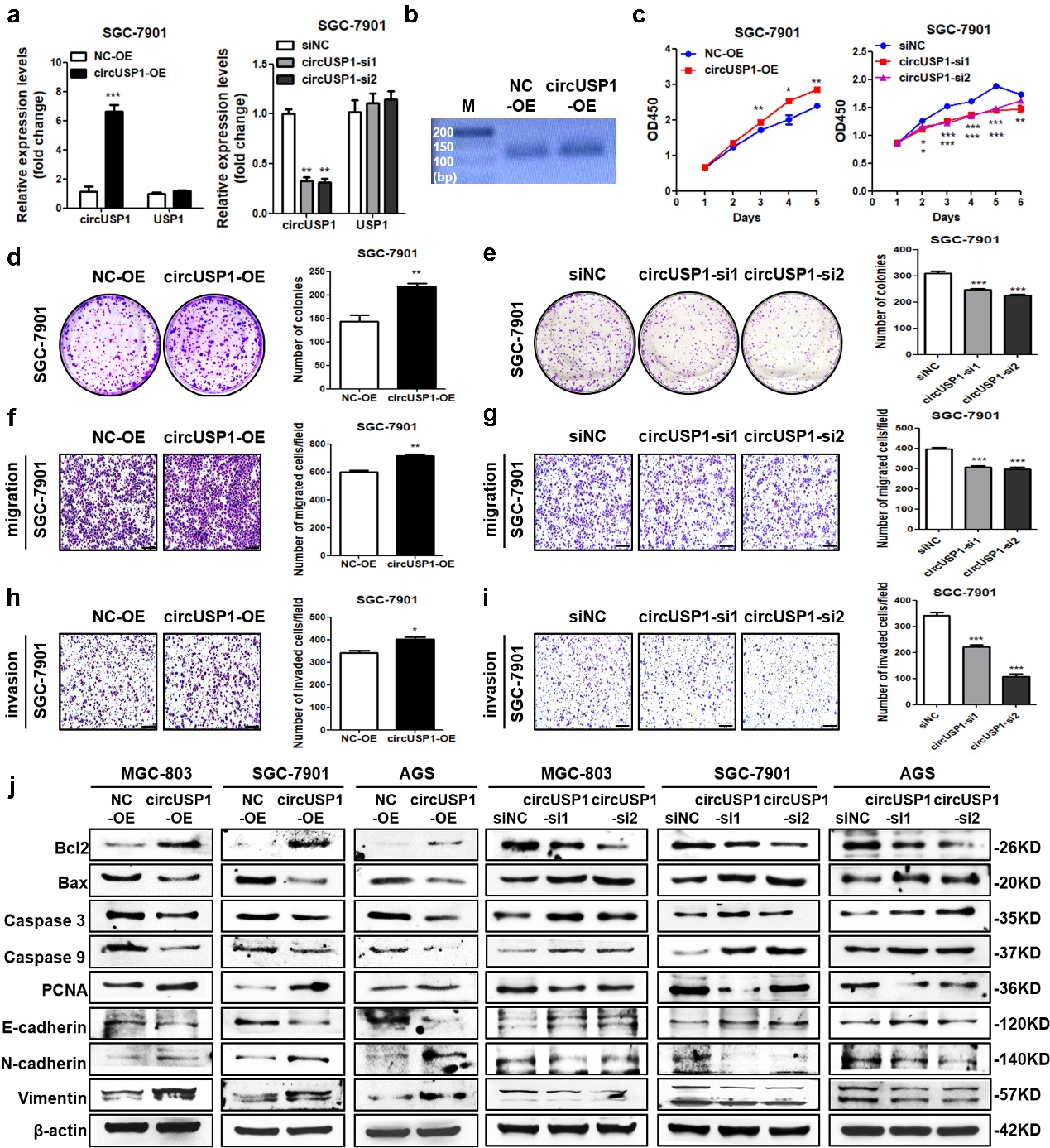


**Fig.S2 CircUSP1 exerts oncogenic effects in GC. a** RT-qPCR analysis of circUSP1 overexpression and knockdown efficiency in SGC-7901 cells. ****p*<0.001 versus NC-OE group, *n*=3 [Student’s t-test]. ***p*<0.01 versus siNC group, *n*=3 [one-way ANOVA followed by Dunnett’s post-hoc test]. **b** Agarose gel electrophoresis analysis of RT-qPCR products of circUSP1 (120bp) in circUSP1-overexpressing MGC-803 cells and its corresponding controls. CCK8 assays (**c**), colony formation assays (**d-e**), migration (**f-g**) and invasion assays (**h-i**) of SGC-7901 cells with circUSP1 overexpression and knockdown. The bar graphs show the quantitative comparison of colony numbers, migrated and invaded cell numbers per field. The scale bar indicates 200 μm. **p*<0.05, ***p*<0.01 versus NC-OE group, *n*=3 [Student’s t-test]. **p*<0.05, ***p*<0.01, ****p*<0.001 versus siNC group, *n*=3 [one-way ANOVA followed by Dunnett’s post-hoc test]. (**j**) Western blot analysis for proliferation-, apoptosis- and EMT-related proteins in three GC cell lines after circUSP1 overexpression and knockdown.


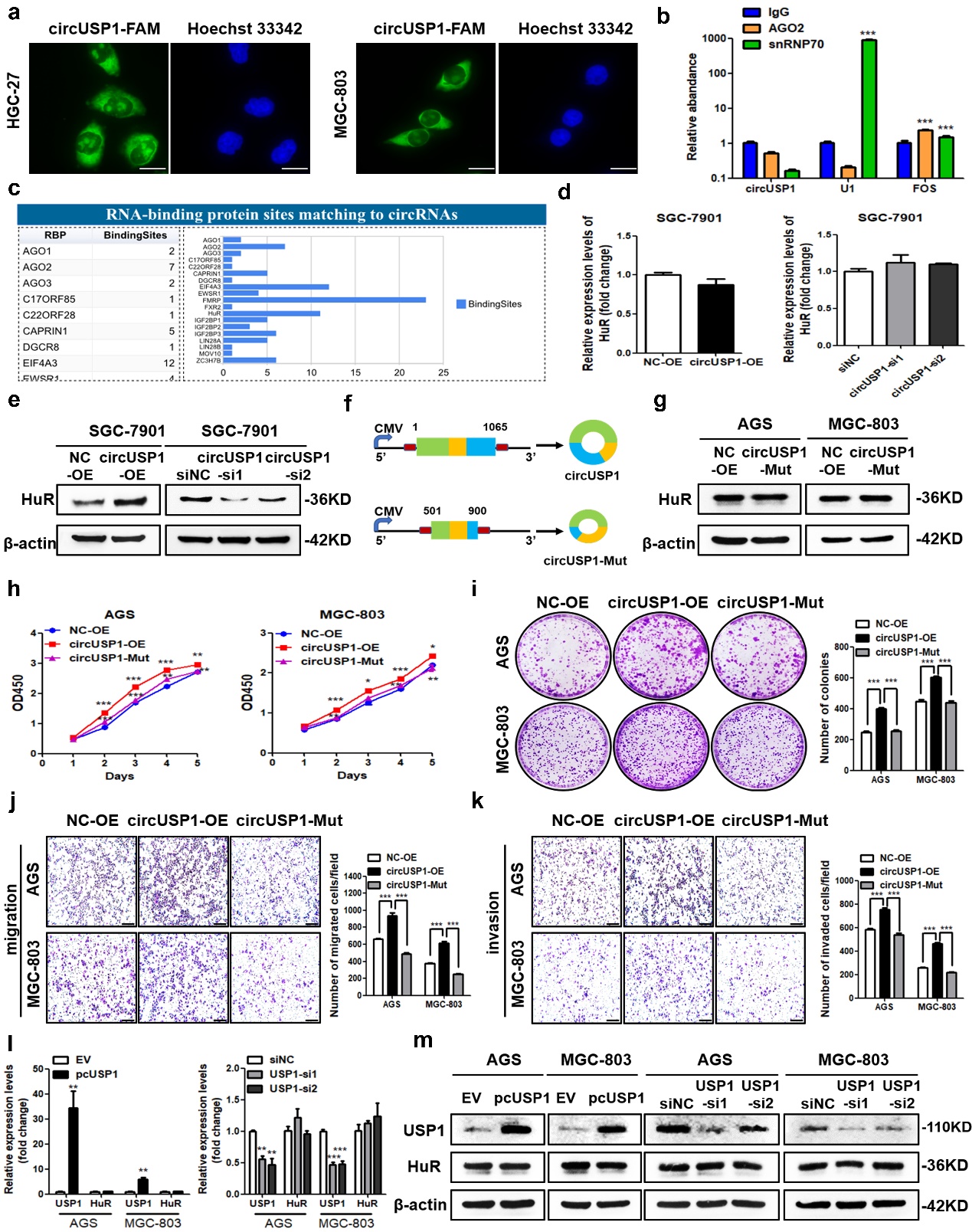


**Fig.S3** **CircUSP1 interacts with HuR and inhibits its ubiquitination and degradation.** **a** RNA FISH analysis of circUSP1 in HGC-27 and MGC-803 cells with specific probes targeting back-spliced junction sites (labeled in green). Nuclei were counterstained with Hoechst 33342 (Blue). Scar bar=10 μm. **b** RIP analysis of circUSP1 in MGC-803 cells using anti-AGO2 antibodies. FOS and U1 were used respectively as the positive control for anti-AGO2 and ani-snRNP70 antibodies. ****p*<0.001 versus lgG group, *n*=3 [one-way ANOVA followed by Dunnett’s post-hoc test]. **c** The potential RBPs interacting with circUSP1 were predicted and shown by circInteractome database. **d-e** RT-qPCR and western blot analyses of HuR mRNA and protein levels after circUSP1 overexpression and knockdown in SGC-7901 cells. **f** Schematic illustration of circUSP1 mutant vector. The flanking circularization elements are marked in red. **g** western blot analysis of HuR protein level after transfection with circUSP1 mutant vector in AGS and MGC-803 cells. CCK8 assays (**h**), colony formation assays (**i**), migration (**j**) and invasion assays (**k**) of AGS and MGC-803 cells after transfection with circUSP1 mutant vector. The bar graphs show the quantitative comparison of colony numbers, migrated and invaded cell numbers per field. The scale bar indicates 200 μm. ****p*<0.001 versus circUSP1-OE group, *n*=3 [one-way ANOVA followed by Bonferroni’s post-hoc test]. **l-m** RT-qPCR and western blot analyses of HuR mRNA and protein levels after USP1 overexpression and knockdown in AGS and MGC-803 cells. ***p*<0.01 versus EV group, *n*=3 [Student’s t-test]. ***p*<0.01, ****p*<0.001 versus siNC group, *n*=3 [one-way ANOVA followed by Dunnett’s post-hoc test].


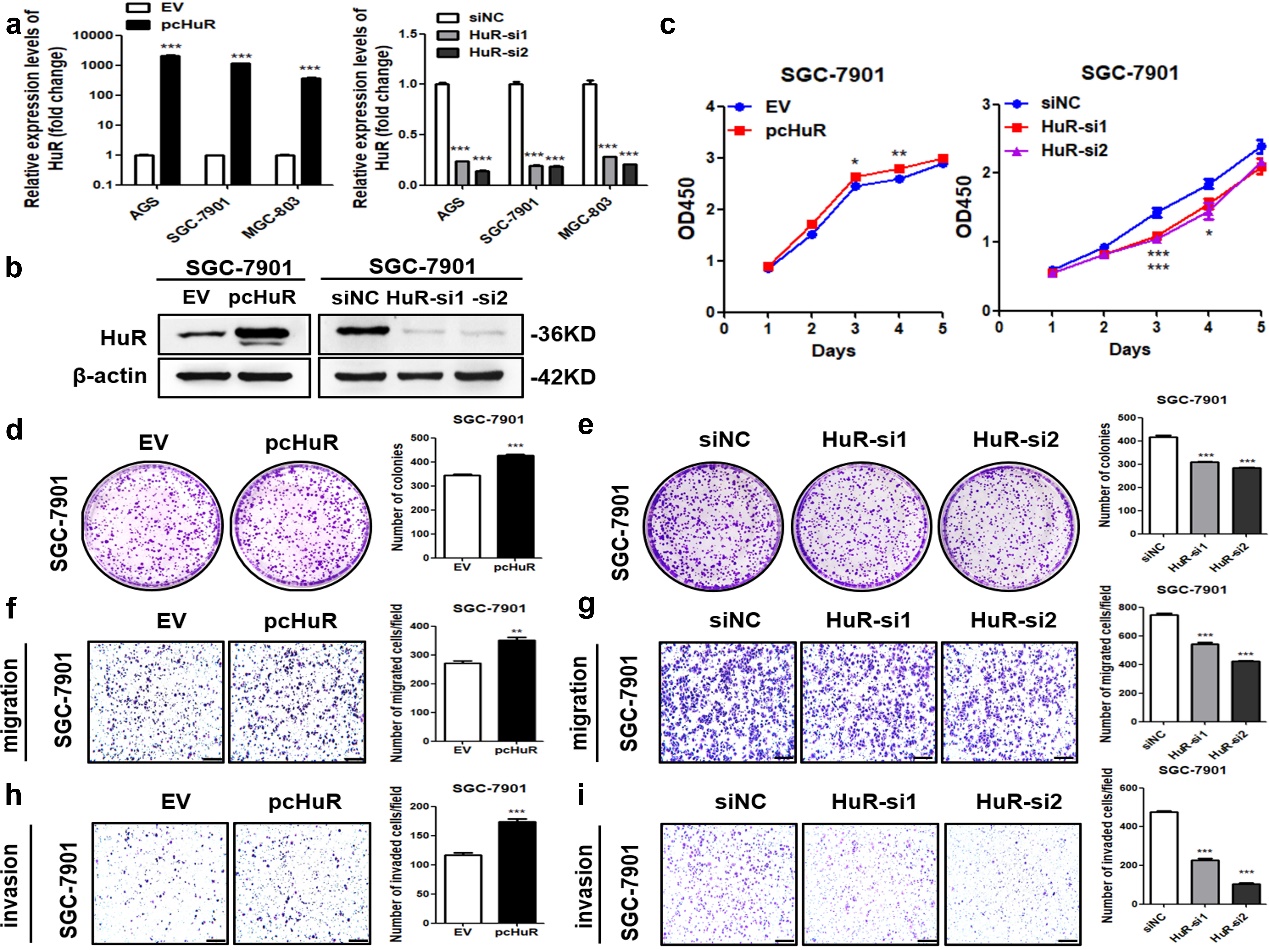


**Fig.S4 HuR mediates the oncogenic effects of circUSP1 in GC progression. a** RT-qPCR analysis for the transfection efficiency of HuR overexpression vector and siRNAs in three GC cell lines. ****p*<0.001 versus EV group, *n*=3 [Student’s t-test]. ****p*<0.001 versus siNC group, *n*=3 [one-way ANOVA followed by Dunnett’s post-hoc test]. **b** Western blot analysis of HuR protein level in SGC-7901 cells after its overexpression and knockdown. CCK8 assays (**c**), colony formation assays (**d-e**), migration (**f-g**) and invasion assays (**h-i**) of SGC-7901 cells with HuR overexpression and knockdown. The scale bar indicates 200 μm. The bar graphs show the quantitative comparison of colony numbers, migrated and invaded cell numbers per field. **p*<0.05, ***p*<0.01, ****p*<0.001 versus EV group, *n*=3 [Student’s t-test]. **p*<0.05, ****p*<0.001 versus siNC group, *n*=3 [one-way ANOVA followed by Dunnett’s post-hoc test].


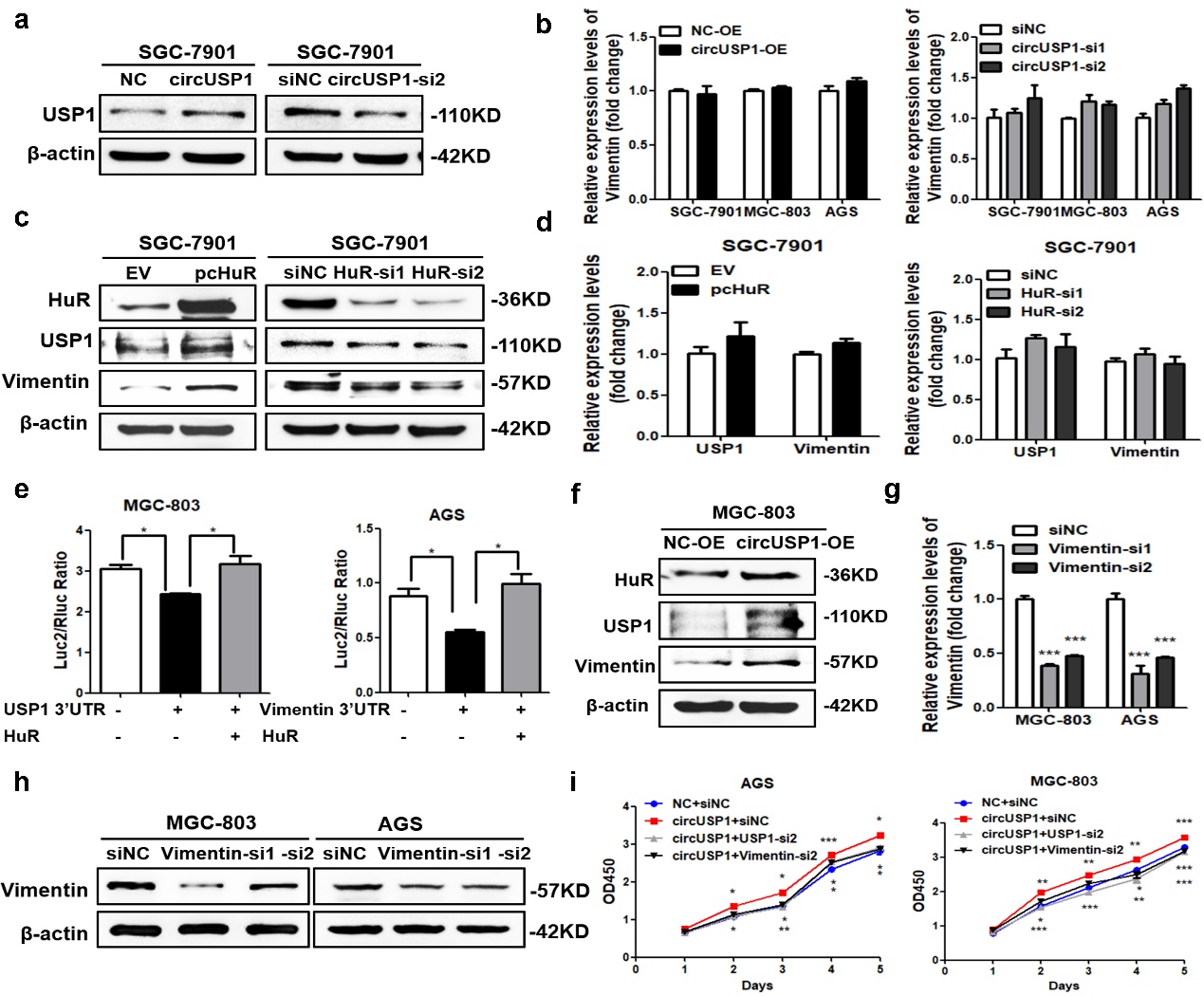


**Fig.S5** **CircUSP1 increases USP1 and Vimentin expression via HuR-mediated post-transcriptional regulation.** **a-b** Western blot analysis of USP1 protein level and RT-qPCR analysis of Vimentin mRNA level after circUSP1 overexpression and knockdown in GC cells. **c-d** Western blot and RT-qPCR analyses for USP1 and Vimentin levels after HuR overexpression and knockdown in SGC-7901 cells. **e** The regulatory effects of HuR on 3’ UTR in USP1 and Vimentin mRNA evaluated by dual-luciferase reporter gene assays. **p*<0.05 versus pmirGLO-USP1 3’ UTR+EV group, *n*=3 [one-way ANOVA followed by Bonferroni’s post-hoc test]. **p*<0.05 versus pmirGLO-Vimentin 3’ UTR+EV group, *n*=3 [one-way ANOVA followed by Bonferroni’s post-hoc test]. **f** The protein levels of HuR, USP1 and Vimentin expression in resected tumor tissues detected by western blot. **g-h** RT-qPCR and western blot analyses of Vimentin knockdown efficiency in two GC lines. ****p*<0.001 versus siNC group, *n*=3 [one-way ANOVA followed by Dunnett’s post-hoc test]. **i** CCK8 assays of circUSP1-overexpressing GC cells co-transfected with USP1 and Vimentin siRNAs. **p*<0.05, ***p*<0.01, ****p*<0.001 versus circUSP1+siNC group, *n*=3 [one-way ANOVA followed by Bonferroni’s post-hoc test].


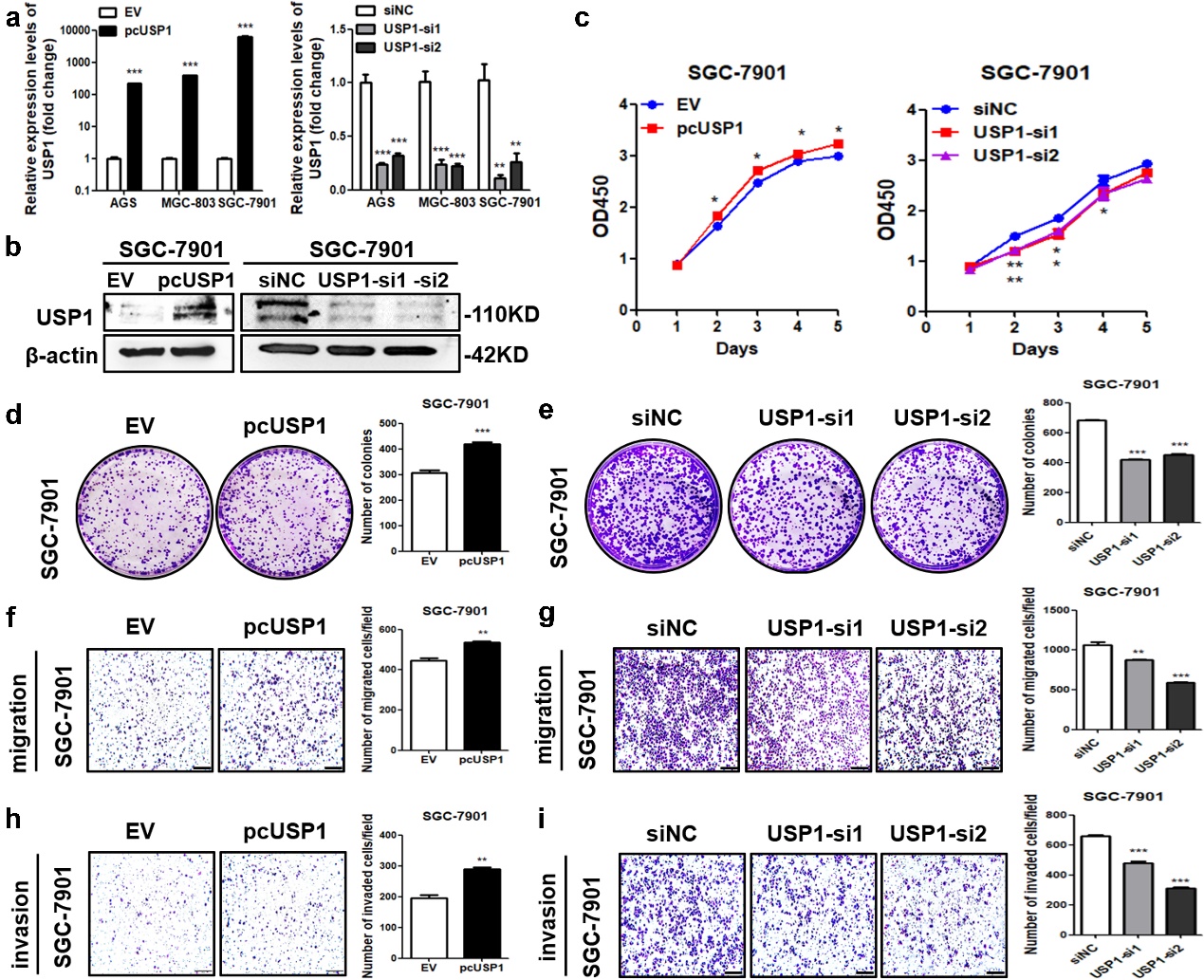


**Fig.S6 USP1 is upregulated in GC and promotes the growth and metastasis of GC cells. a-b** RT-qPCR and western blot analyses for the transfection efficiency of USP1 overexpression vector and siRNAs in GC cell lines. ****p*<0.001 versus EV group, *n*=3 [Student’s t-test]. ***p*<0.01, ****p*<0.001 versus siNC group, *n*=3 [one-way ANOVA followed by Dunnett’s post-hoc test]. CCK8 assays (**c**), colony formation assays (**d-e**), migration (**f-g**) and invasion assays (**h-i**) of SGC-7901 cells with USP1 overexpression and knockdown. The bar graphs show the quantitative comparison of colony numbers, migrated and invaded cell numbers per field. The scale bar indicates 200 μm. **p*<0.05, ***p*<0.01, ****p*<0.001 versus EV group, *n*=3 [Student’s t-test]. **p*<0.05, ***p*<0.01, ****p*<0.001 versus siNC group, *n*=3 [one-way ANOVA followed by Dunnett’s post-hoc test].

**
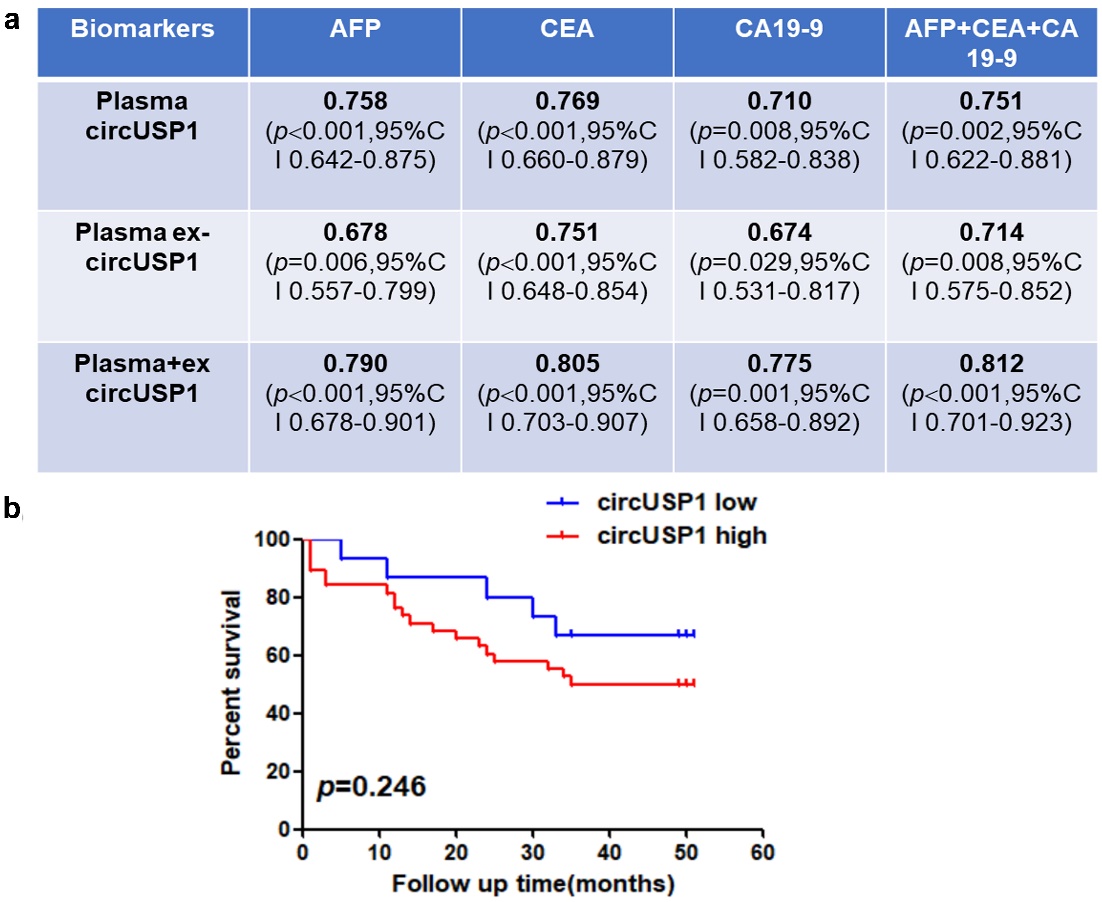
**

**Fig.S7 CircUSP1 in peripheral blood is a potential diagnostic biomarker. a** The AUC values for the different combinations of traditional serum tumor markers (AFP, CEA and CA19-9), plasma- and plasma exosome-derived circUSP1 using ROC curve analysis. **b** Kaplan-Meier analysis of the correlation between circUSP1 level in GC plasma with overall survival (OS) of GC patients.

**Sequence analysis for AREs (used in Fig. 5g)**

**SA1**. 3’UTR of USP1 (NM_001017415.2)

AGTGAGTGTATTTTCCTTGTGTATATATTAAACACACCCATACAAACATTGGTAAAGTTGATTACATCAAAGAATCTTTAGCTTATCTTTTGAAGCTACTGGATATTATTGGTCTCTCTAGGTTTTTATATAAATAGTGAAATTTGAATTACTGAAAACCATGTTAATTTTTAGAACTCATTTTCCTCAGTAGAGACTAGTGATGCATTAGCTTCTGGGAACAAACTTGTATCGGTTCTTAATTAAATTATCCAAAACGGAGGCATTTAAACACTTGGATTTACACCAGTCTTTTGTGTTTGCTTTTTAAAATAAAGTGCTCGTATTTGTATTCTCCATATTTTGGAGTAATTATCTACATGATGTTTATAGTTCCTGTGGTTTTTCACCCAAGAAGCAGAATCTCATTCAGTACATTTAGTTTTATAAGAGTCATGAAGCTAAATCCTTGGGCTATGTCAGAGGCACAAAGTCTAGAATGTGTGTATTCACAATGGTGTATGTACATTTTGTGCCTTGATTCACTTAGAAGTGTCTCAGAAAACCTGGACAGTTCGCTTCTACACAAGAATTTTATATGTATTTATGAAGATGATTCTGTACCCTAGTATATCTTTTTGGGCATGGACTAATTTGTATCTGTTTAACTCATATTCTGCACGATCTGTATATAGTACATCAAACTTAGAGGTGTGACCTTAAATTTAACTTTTTTTAAAAACTGGGAGGTCAATAAAATTTAAACTGCTTAACTATGTATATGAATATTTGAATTTTTTACTTGTATATTTTTATAAATACAGCTGAGTTTTCTTAAAGCGAAAAAAAAAAAA

**SA2**. 3’UTR of Vimentin (NM_003380.5)

AAATTGCACACACTCAGTGCAGCAATATATTACCAGCAAGAATAAAAAAGAAATCCATATCTTAAAGAAACAGCTTTCAAGTGCCTTTCTGCAGTTTTTCAGGAGCGCAAGATAGATTTGGAATAGGAATAAGCTCTAGTTCTTAACAACCGACACTCCTACAAGATTTAGAAAAAAGTTTACAACATAATCTAGTTTACAGAAAAATCTTGTGCTAGAATACTTTTTAAAAGGTATTTTGAATACCATTAAAACTGCTTTTTTTTTTCCAGCAAGTATCCAACCAACTTGGTTCTGCTTCAATAAATCTTTGGAAAAACTC
